# A differentiated digital intervention to improve antiretroviral therapy adherence among men who have sex with men living with HIV in China: a randomized controlled trial

**DOI:** 10.1186/s12916-022-02538-3

**Published:** 2022-10-10

**Authors:** Kedi Jiao, Chunmei Wang, Meizhen Liao, Jing Ma, Dianmin Kang, Weiming Tang, Joseph D. Tucker, Wei Ma

**Affiliations:** 1 Department of Epidemiology, School of Public Health, Cheeloo College of Medicine, Shandong University, Jinan, Shandong People’s Republic of China; 2 Shandong Public Health Clinical Center, Jinan, Shandong People’s Republic of China; 3 Institution for AIDS/STD Control and Prevention, Shandong Center for Disease Control and Prevention, Jinan, Shandong People’s Republic of China; 4 University of North Carolina Chapel Hill Project-China, Guangzhou, Guangdong People’s Republic of China; 5 Clinical Research Department, Faculty of Infectious and Tropical Diseases, London School of Hygiene and Tropical Medicine, London, UK

**Keywords:** Anti-retroviral agents, Therapeutics, Medication adherence, Digital technology, Sexual and gender minorities, Randomized controlled trial

## Abstract

**Background:**

Antiretroviral therapy (ART) adherence is still suboptimal among some key populations, highlighting the need for innovative tailored strategies. This randomized controlled trial (RCT) aimed to evaluate the effect of a differentiated digital intervention on ART adherence among men who have sex with men (MSM) living with HIV in China.

**Methods:**

The two-armed parallel RCT was conducted at one HIV clinic in Jinan of China from October 19, 2020, to June 31, 2021. Men were referred by health providers to join the study and then choose one of three digital strategies—text message, only instant message, or instant message plus social media. They were assigned in a 1:1 ratio to the intervention arm or control arm using block randomization, and inside each arm, there were three groups depending on the type of delivering the message. The groups were divided according to participants’ preferred digital strategies. The intervention arm received ART medication messages, medication reminders, peer education, and involved in online discussion. The control arm received messages on health behavior and nutrition. The primary outcome was self-reported optimal ART adherence, defined as not missing any doses and not having any delayed doses within a one-month period. Secondary outcomes included CD4 T cell counts, viral suppression, HIV treatment adherence self-efficacy, and quality of life. Intention-to-treat analysis with generalized linear mixed models was used to evaluate the intervention’s effect.

**Results:**

A total of 576 participants were enrolled, including 288 participants assigned in the intervention arm and 288 assigned in the control arm. Most were ≤ 40 years old (79.9%) and initiated ART ≤ 3 years (60.4%). After intervention, the proportion of participants achieving optimal ART adherence in the intervention arm was higher than in the control arm (82.9% vs 71.1%). The differentiated digital intervention significantly improved ART adherence (RR = 1.74, 95%CI 1.21–2.50). Subgroup analysis showed one-to-one instant message-based intervention significantly improved ART adherence (RR = 2.40, 95% CI 1.39–4.17).

**Conclusions:**

The differentiated digital intervention improved ART adherence among MSM living with HIV in China, which could be integrated into people living with HIV (PLWH) management and further promoted in areas where PLWH can access text messaging and instant messaging services.

**Trial registration:**

ChiCTR2000041282. Retrospectively registered on 23 December 2020.

**Supplementary Information:**

The online version contains supplementary material available at 10.1186/s12916-022-02538-3.

## Background

The treat-all strategy has allowed most PLWH to start antiretroviral therapy (ART), and on this circumstance, improving the adherence is essential in achieving the target of treat-all [1, 2]. By the end of June 2021, about 28.2 million people living with HIV (PLWH) globally were on ART [3]. However, suboptimal ART adherence has been reported in both high-income countries [4, 5] and low- and middle-income countries (LMICs), including China [6, 7]. The reasons of suboptimal adherence are diverse and include individual, inter-personal and structural factors [8, 9].

Men who have sex with men (MSM) are the key populations who are particularly vulnerable to HIV infection and present an increasing public health challenge. In 2020, MSM accounted for 45% of HIV infections outside of sub-Saharan Africa [10]. In China, the proportion of male-to-male transmission increased from 9.1% in 2009 to 23.3% in 2020 [11]. In Shandong Province of China, male-to-male transmission accounted for 57.3% (6195/10808) of all HIV/AIDS cases reported from 1992 to 2016 [12]. Stigma associated with taking ART, limited access to HIV care, and diminished trust in health providers may be particularly important in driving poor ART adherence among MSM living with HIV [13, 14]. For instance, less than 80% of Hispanic/Latino MSM in the USA reported high level of ART adherence [13]. And furthermore, only about 60% of all MSM living with HIV were estimated to achieve viral suppression in US [9]. In Kenya, 40% of MSM had less than 95% adherence (versus 28.6% of heterosexual men) [14]. Nevertheless, few specific adherence interventions focused on MSM, highlighting the need for tailored and innovative strategies to enhance ART adherence in this population [15]. Due to convenience and confidentiality, digital technology may be an important tool to reach the marginalized group [16].

Digital health interventions are defined as interventions using information and communication technology to support health [17]. Digital innovations can be further divided into non-Internet (e.g., text message/short message service [SMS] and phone calls) and Internet-based (e.g., social media, mobile application and website) technologies [18]. Internet-based digital interventions allow users to generate content and disseminate information on sensitive topics at any time or place and can potentially decrease inadvertent disclosure of sensitive behaviors [19–21]. The global expansion of instant messaging platforms has provided a foundation for Internet-based digital intervention in recent years. For example, one instant messaging service (WhatsApp) has two billion monthly active users globally and is common in 180 countries, with options that do not require a smartphone [22]. However, few randomized controlled trials (RCT) have assessed Internet-based digital interventions to improve ART adherence [16]. By contrast, SMS has been widely used to deliver adherence interventions and is already recommended by the World Health Organization and others [23–26].

Although many strategies have been used to improve ART adherence, much is still not well known about optimizing ART adherence. A meta-analysis showed that multiple strategies were superior to a single strategy, suggesting the importance of multi-component interventions [27]. The World Health Organization and the Joint United Nations Programme on HIV/AIDS (UNAIDS) have highlighted the need for differentiated care across the HIV care continuum [10, 23]. Differentiated care is defined as a patient-centered approach adapting HIV services to reflect the preferences and expectations of PLWH [28]. The central driver of differentiated care is patients’ need. Differentiated care can improve adherence, increase patients’ satisfaction, empower patients, and make health systems more efficient [28]. Duncombe et al. developed a differentiated framework for HIV treatment services with variations in type of services delivered, service frequency, health worker cadre, and service location [29]. Despite the known importance of patient-centered approaches, few studies have differentiated ART adherence strategies [30].

This RCT aimed to evaluate a differentiated digital intervention to improve ART adherence among MSM living with HIV in China.

## Methods

### Trial design

**Fig. 1:**
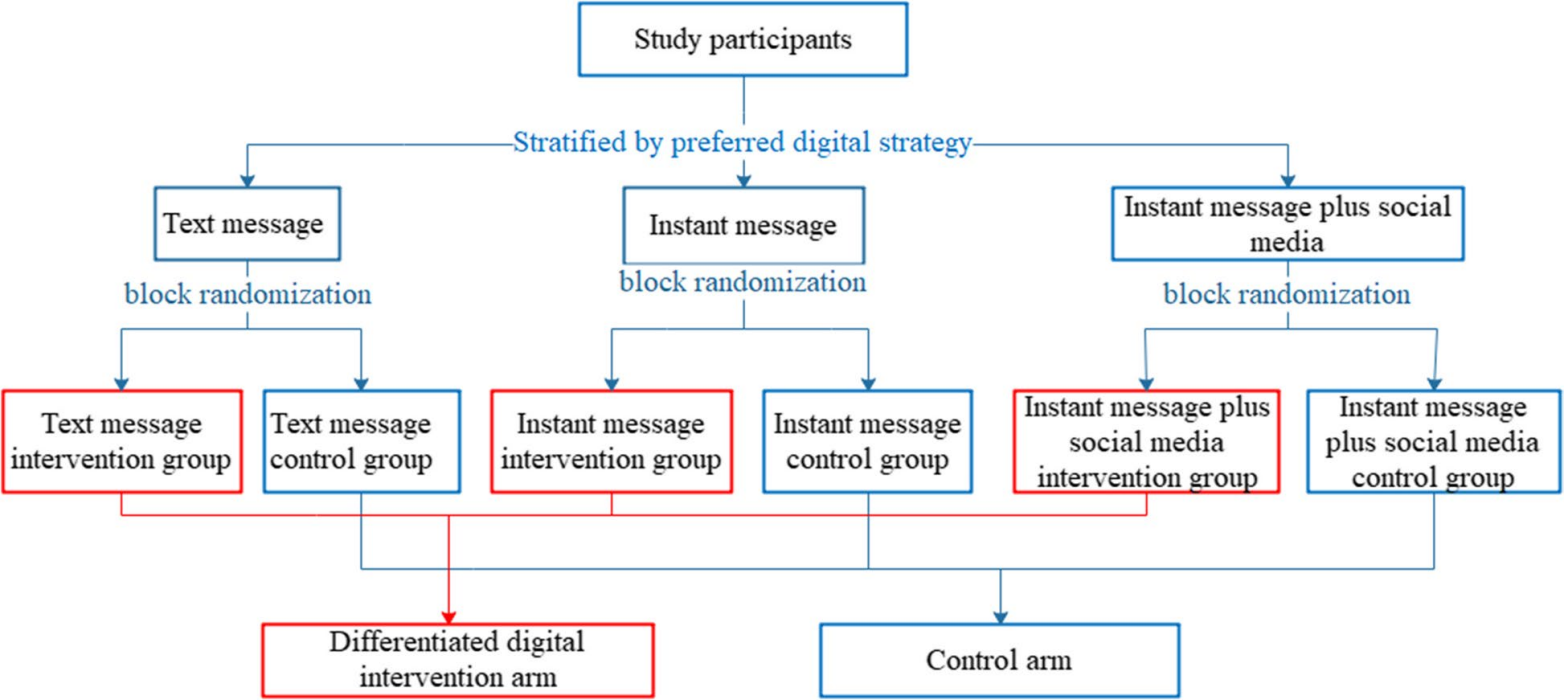
Stratified block randomization profile

This is a pragmatic two-armed parallel RCT, and inside each arm, there were three groups depending on the type of delivering the message and the type of information (see Fig. 1). According to the study design, participants were allowed to choose one of the three digital strategies (text message, only instant message, and instant message plus social media) based on their preferences. Then, they were assigned in a 1:1 ratio to the intervention arm or control arm using stratified block randomization. Stratification was according to participants’ preferred digital strategy and block randomization was triggered within each stratum. We used WeChat for instant messaging and QQ for social media. Both of these Chinese platforms are common instant messaging platforms similar to Facebook and Twitter, respectively. This RCT included a baseline survey and two follow-ups with 3-month interval. More details about study design were displayed in study protocol (see Additional file 1). We followed the standard guidelines for reporting parallel group RCTs (see Additional file 2) [31].

### Participants

This RCT was conducted in an infectious diseases hospital in Jinan, Shandong Province of China from October 19, 2020 to June 31, 2021. This hospital is a tertiary referral site that provides ART and post-exposure prophylaxis (PEP) for approximately 1600 MSM (accounting for about 80% of all patients). All patients need to visit their health care providers at least every 3 months for medication refills and physical examinations.

Eligible participants were aged 18 years or above, living with HIV and currently on ART in the hospital, born biologically male, having ever reported anal sex with men, and willing to choose one of the three provided digital strategies. Individuals were excluded if they did not have their own mobile phones or could not access Internet or could not complete the survey due to personal reasons (e.g., psychiatric disorders). Participants were recruited when they came to refill medication. The health providers first screened the eligibility of each patient and referred eligible people to a waiting room where trained investigators further identified their eligibility and provided a detailed introduction of the study. Those who showed interest, met eligibility criteria, and provided written informed consent were asked to complete a baseline survey. At the same time, they were required to provide their phone number or WeChat account according to their preferred digital strategy. Thereafter, they were asked to complete two follow-up surveys every three months when they came to refill ART medications. Men were reimbursed about 7.85 USD for each of the survey questionnaires completed.

### Interventions

The development of intervention was guided by the theory of planned behavior [32], a scoping literature review on digital ART adherence interventions [16, 23, 27, 33, 34], and formative research focused on barriers and facilitators of digital interventions (see details in Additional file 1).

**Fig. 2:**
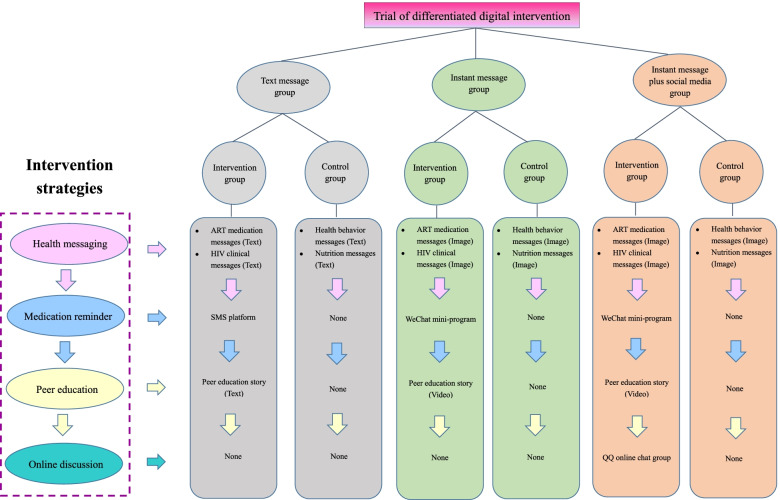
Differentiated digital intervention profile

The final comprehensive interventions included health messaging (ART medication messages and HIV clinical messages), medication reminder, peer education, and online group discussion. For the control arm, participants also received additional messages (health behavior and nutrition messages) based on routine care. According to the study design, there were three groups within each arm depending on the type of delivering the message and the type of information. For instance, we delivered health messages using text in text message group but using images in instant message group (see Fig. 2).

In the text message-based intervention group, we delivered ART medication messages (12 items in total, Additional file 3: Table S1) biweekly and HIV clinical messages (6 items in total, Additional file 3: Table S2) monthly via text message. After the first follow-up, we additionally delivered peer education stories (6 items in total, Additional file 3: Table S3) biweekly via text message. Daily medication reminders were sent to those who needed the service at 3 min before their dose-timing via a customized SMS platform.

In the instant message-based intervention group, we delivered images of ART medication messages (https://github.com/JKD-epidemiology/Intervention-materials.git) biweekly and HIV clinical messages (https://github.com/JKD-epidemiology/Intervention-materials.git) monthly via WeChat. After the first follow-up, we additionally delivered peer education videos (https://github.com/JKD-epidemiology/Intervention-materials.git) biweekly via WeChat. The images and videos were designed and recorded by peer volunteers from a MSM community-based organization. A WeChat mini-program (a lightweight “APP” without the need of downloading) was developed to provide daily medication reminders and online clock punching after taking medication (see user interface in https://github.com/JKD-epidemiology/Intervention-materials.git) [35]. Participants who were interested could enter the mini-program to complete clock punching within one hour of prescribed dose-timing. Those who completed 30-day uninterrupted clock punching could receive about 4.71 USD as a reward and this was cumulative during the intervention periods. In addition, the mini-program sent daily medication reminders to those who needed the service at 3 min  before their dose-timing.

In the instant message plus social media intervention group, we additionally established a QQ group. A project member (as group administrator) and a health provider were invited to the QQ group to encourage participants to communicate about ART experience and share health messaging. In addition, the administrator shared short articles (24 items in total, Additional file 4) about frontiers of HIV research and tips of ART to this group weekly, which were adapted from authoritative website (e.g., UNAIDS, China CDC).

In the control arm, we delivered messages about health behavior and nutrition (12 items in total, Additional file 3: Table S4) biweekly based on routine care provided by the local hospital and CDC. We also delivered messages according to participants’ preferred digital strategies. Specifically, we delivered text messages for text message control group and images (https://github.com/JKD-epidemiology/Intervention-materials.git) for instant message control group and instant message plus social media control group.

### Outcomes

The primary outcome was the proportion of achieving optimal ART adherence over the previous month, which was assessed based on self-report at baseline and each follow-up. ART adherence had two components: percent adherence (percent of prescribed doses taken) and dose-timing adherence (how many hours it was taken is away from the recommended time for the particular antiretroviral medication). We defined optimal ART adherence as not missing any doses and not having any delayed doses (more than 1 h) within a month period. The 1-h window of dose-timing adherence was based on clinical suggestion and a digital intervention study in China [36]. The secondary outcome included proportions of viral suppression (defined as viral load < 20 copies/ml), CD4 T cell counts (cells/mm^3^), HIV treatment adherence self-efficacy, and quality of life. We collected CD4 T cell counts and HIV viral load from medical records. We adapted validated scales to measure HIV treatment adherence self-efficacy [37] and quality of life [38]. Detailed introduction of the scales were displayed in study protocol (Additional file 1). The self-efficacy and quality of life were measured at baseline and in each follow-up survey. The designated ART hospital regularly provides free CD4 T cells and viral load testing for all patients from March to June every year, the time of which is basically consistent with our second follow-up. We collected data in 2020 as baseline (pre-intervention) measurement and data in 2021 as post-intervention measurement.

### Sample size

We first calculated a sample size based on our pre-specified subgroup analyses by different digital strategies and then extrapolated the total sample size according to the estimated proportions of joining each subgroup. We assumed a fifteen-percentage difference in proportions of achieving optimal ART adherence post-intervention for the text message subgroup (95% in intervention group vs. 80% in control group) based on a previous SMS intervention in Chinese context [39]. Assuming two-sided alpha = 0.05, 80% power, and 20% loss to follow-up, we calculated the target sample size of text message subgroup was 190. For the instant message and instant message plus social media subgroups, there was no preliminary data, and we estimated that the percentage difference of achieving optimal adherence was no less than fifteen.

A pilot survey in another designated ART delivery site of Shandong Province showed two-thirds of PLWH would prefer instant message and the remaining one third preferred text message. Accordingly, we anticipated that one-third of participants would choose text message in this study and extrapolated the total sample size was 570.

### Randomization and blinding

We randomly assigned the participants in a 1:1 ratio to either the intervention arm or control arm with a stratified block design (block sizes of four). Stratification was according to participants’ preferred digital strategy (*n* = 3). A research assistant with no involvement in the trial generated the computerized random allocation sequence and performed packaging of sequentially numbered, opaque, sealed envelopes. The health providers and investigators who were masked to random allocation sequence participated in enrolling participants. The investigators who were independent of outcome assessment assigned participants to interventions. The allocation sequence was applied to participants sequentially in the order in which they were enrolled within the group they chose.

Due to study design, participants and intervention providers were aware of the assigned arm. Those assessing outcomes, field investigators, and health providers who referred participants to this study were kept blind to assignment.

### Statistical analysis

Descriptive analysis was used to summarize basic characteristics. We also described and compared the proportions of participants achieving optimal ART adherence between the intervention arm and control arm at baseline and each follow-up. We evaluated the effect of intervention on primary and secondary outcomes with intention-to-treat (ITT) analysis using generalized linear mixed models (GLMMs). For GLMMs, the intervention status, stratification status, time indicators, and baseline outcome (to control for potential imbalance after randomization) were regarded as fixed effects, while individuals with multiple measurements were regarded as random effects. Further, we evaluated the intervention effect adjusted for age, education, and ART duration. The estimated intervention effects (risk ratio [RR] and absolute risk difference [RD] for binary outcomes and mean difference [MD] for continuous outcomes) were reported with 95% confidence intervals (CI). Subgroup analysis by different digital strategies (text message versus instant message versus instant message plus social media group) was conducted to evaluate the primary and secondary outcomes between the intervention group and control group within each stratum. Additional subgroup analysis by ART duration (≤ 3 years versus > 3 years) was conducted to evaluate the difference in proportion of optimal ART adherence between the intervention arm and control arm based on pre-specified analysis plan.

We conducted sensitivity analysis using per-protocol (PP) strategy and as-treated (AT) strategy to evaluate the stability of intervention and effect of contamination. In PP analysis, we encountered non-convergence phenomenon and used log Poisson GLMM to substitute log binomial GLMM. Given baseline differences in the ART adherence in the instant message subgroup, we additionally conducted sensitivity analysis by combining two subgroups (i.e., instant message subgroup and instant message plus social media subgroup) to evaluate the effectiveness of instant message-based intervention. According to our pre-specified analysis plan, we used complete-case analysis without adopting multiple imputations because the missing of outcome variable was < 15%.

Descriptive analysis was conducted using SPSS 24.0. The GLMMs were conducted using SAS 9.4.

### Ethical approval

This study was performed in accordance with the Declaration of Helsinki and approved by the Ethical Review Committee of School of Public Health in Shandong University (20190210). Written informed consent were obtained from all participants. All investigators, intervention providers, and assessors signed non-disclosure protocols before recruitment. The trial was registered in the Chinese Clinical Trial Registry (ChiCTR2000041282).

## Results

### Trial profile

Men were recruited from October 19, 2020, to December 4, 2020, and followed until June 31, 2021. Among 593 people referred by health providers, 586 were eligible and completed the baseline survey. Among those who were excluded, three men reported never having anal sex with men, two men were not interested or refused to provide informed consent, and two had already enrolled once in the study. Among eligible participants, nine people refused to provide a phone number or WeChat account after completing baseline survey, and one dropped out before randomization. A total of 576 participants were enrolled and randomized. Among this group, 144 (25.0%) chose text messages, 290 (50.3%) chose instant messages, and 142 (24.7%) chose instant messages plus social media. Based on the predetermined randomization list, stratified block randomization was performed, and 288 participants (including 72 in text message intervention group, 145 in instant message intervention group, 71 in instant message plus social media intervention group) were assigned to the intervention arm and 288 (including 72 in text message control group, 145 in instant message control group, 71 in instant message plus social media control group) were assigned to the control arm.

**Fig. 3:**
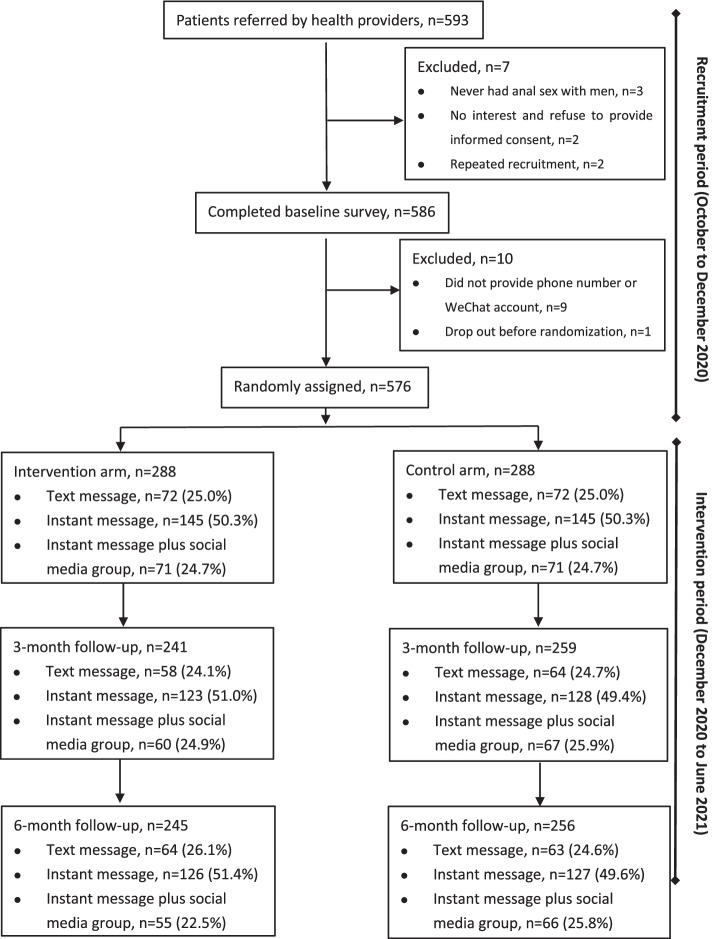
Trial profile

A total of 500 participants completed the 3-month follow-up survey with a loss-to-follow-up rate of 13.2% (76/576) and 501 completed the 6-month follow-up survey with a loss-to-follow-up rate of 13.0% (75/576). The reasons of lost to follow-up mainly included that participants were reluctant to participate in this study or health providers forgot referral of the follow-up surveys. A total of 547 participants (95.0%) completed as least one follow-up and were included in ITT analysis (including 268 participants in the intervention arm and 279 participants in the control arm) (Fig. 3).

### Baseline characteristics of the participants

As displayed in Table 1, participants’ sociodemographic, behavioral, and clinical characteristics in the intervention arm and control arm were similar. The majority of participants were 40 years old or younger (79.9%), had a college degree or beyond (61.3%), had an annual income below 15158 USD (78.6%), and were unmarried/divorced/widowed (80.7%). About 60% of participants reported their sexual orientation as gay. 55.9% and 62.3% of participants had disclosed their sexual orientation and HIV status to others. More than half of the participants (58.3%) reported alcohol use in the past 12 months.

**Table 1:** Sociodemographic, behavioral, and clinical characteristics of the study participants in a randomized controlled trial of differentiated digital intervention in China, 2020–2021

Characteristics	Total (***N*** = 576)	Intervention arm (***n*** = 288)	Control arm (***n*** = 288)
**Age, years**			
≤ 30	214 (37.2)	101 (35.1)	113 (39.2)
31–40	246 (42.7)	128 (44.4)	118 (41.0)
> 40	116 (20.1)	59 (20.5)	57 (19.8)
**Education level**			
High school or below	222 (38.5)	118 (41.0)	104 (36.1)
College or beyond	353 (61.3)	169 (58.7)	184 (63.9)
Missing value	1 (0.2)	1 (0.3)	0 (0.0)
**Annual income, USD**			
≤ 9474	276 (47.9)	148 (51.4)	128 (44.4)
9475–15158	177 (30.7)	82 (28.5)	95 (33.0)
> 15158	123 (21.4)	58 (20.1)	65 (22.6)
**Marital status**			
Unmarried/divorced/widowed	465 (80.7)	221 (76.7)	244 (84.7)
Married/cohabitating	111 (19.3)	67 (23.3)	44 (15.3)
**Sexual orientation**			
Gay	348 (60.4)	164 (56.9)	184 (63.9)
Bisexual	151 (26.2)	80 (27.8)	71 (24.7)
Heterosexual or others	77 (13.4)	44 (15.3)	33 (11.4)
**Sexual orientation disclosure**^**a**^			
Yes	322 (55.9)	169 (58.7)	153 (53.1)
No	254 (44.1)	119 (41.3)	135 (46.9)
**HIV status disclosure**^**b**^			
Yes	359 (62.3)	181 (62.8)	178 (61.8)
No	217 (37.7)	107 (37.2)	110 (38.2)
**Alcohol use in the past 12 months**			
Yes	336 (58.3)	163 (56.6)	173 (60.1)
No	240 (41.7)	125 (43.4)	115 (39.9)
**Viral suppression**^**c**^			
Yes	432 (75.0)	209 (72.6)	223 (77.4)
No	79 (13.7)	46 (16.0)	33 (11.5)
Missing value	65 (11.3)	33 (11.4)	32 (11.1)
**CD4 T cell counts, M±IQR**	584.50±385.00	586.00±434.00	583.00±352.50
**Duration of ART, years**			
≤ 3	348 (60.4)	177 (61.5)	171 (59.4)
> 3	228 (39.6)	111 (38.5)	117 (40.6)
**Frequency of medication-taking**			
Once a day	331 (57.4)	165 (57.3)	166 (57.6)
Twice a day	244 (42.4)	122 (42.4)	122 (42.4)
Missing value	1 (0.2)	1 (0.3)	0 (0.0)
**Regime of ART**			
Three	531 (92.2)	265 (92.0)	266 (92.4)
Two or one	45 (7.8)	23 (8.0)	22 (7.6)
**Received adherence education before ART initiation**			
Yes	545 (94.6)	274 (95.1)	271 (94.1)
No	31 (5.4)	14 (4.9)	17 (5.9)
**Used medication reminders**			
Yes	527 (91.5)	261 (90.6)	266 (92.4)
No	49 (8.5)	27 (9.4)	22 (7.6)
**Ever experienced any side effects during ART**			
Yes	357 (62.0)	180 (62.5)	177 (61.5)
No	219 (38.0)	108 (37.5)	111 (38.5)

Data are presented as no. (%) unless otherwise indicated

*Abbreviations*: *ART* antiretroviral therapy, *USD* United States dollars, *IQR* interquartile range

^a^Has told anyone (except homosexual partner) about sexual orientation or sexual history with men

^b^Has told anyone (except health providers in designated ART sites) about HIV positive-status

^c^Defined as undetectable viral load (i.e., < 20 copies/ml)

The proportion of virally suppressed was 84.5% (missing values were not taken into account) and the median of CD4 T cell counts was 584.50 ± 385.00. In addition, 60.4% of participants initiated ART ≤ 3 years, 57.4% took ART medications once a day, and 62.0% experienced side effects during ART. Most participants took three kinds of antiretroviral medication (92.2%), received adherence education before ART initiation (94.6%), and used medication reminders (91.5%).

The basic characteristics of three groups within each arm were displayed in Table S1 of Additional file 5.

### Proportion of achieving optimal ART medication adherence

**Fig. 4:**
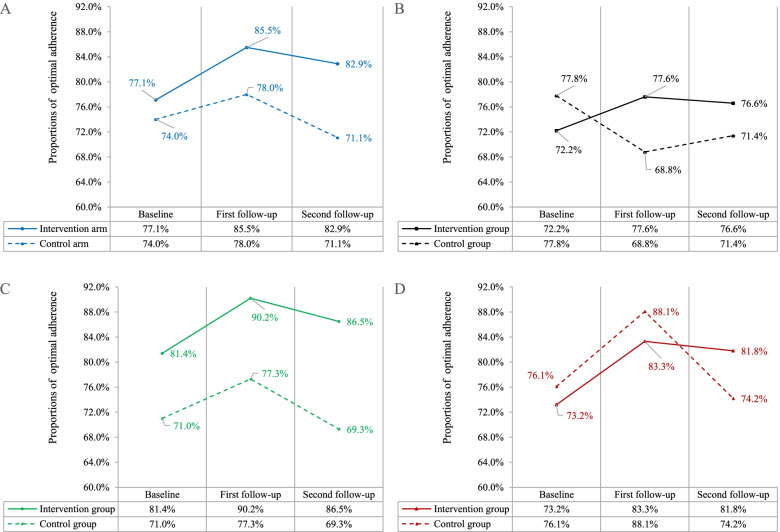
Proportions of achieving optimal ART adherence at baseline and two follow-ups. **A** Proportions of optimal ART adherence in total. **B** Proportions of optimal ART adherence in text message subgroup. **C** Proportions of optimal ART adherence in instant message subgroup. **D** Proportions of optimal ART adherence in instant message plus social media subgroup. ART, antiretroviral therapy

At baseline, the proportion of optimal ART adherence was 77.1% in the intervention arm (72.2% in text message intervention group; 81.4% in instant message intervention group; 73.2% in instant message plus social media intervention group) and 74.0% in the control arm (77.8% in text message control group; 71.0% in instant message control group; 76.1% in instant message plus social media control group) (Fig. 4).

At the second follow-up (post-intervention), the proportion of optimal ART adherence was 82.9% in the intervention arm (76.6% in text message intervention group; 86.5% in instant message intervention group; 81.8% in instant message plus social media intervention group) and 71.1% in the control arm (71.4% in text message control group; 69.3% in instant message intervention group; 74.2% in instant message plus social media intervention group) (Fig. 4).

The measurements of secondary outcomes during the study periods are shown in Table S1-S3 of Additional file 6.

### Effect of differentiated digital intervention on ART adherence

The ITT analysis showed that the differentiated digital intervention significantly improved the proportions of optimal ART adherence (RR = 1.74, 95% CI 1.21–2.50). Subgroup analysis by ART duration showed that the differentiated digital intervention was effective among those who initiated ART ≤ 3 years (RR = 1.75, 95% CI 1.08–2.84). Subgroup analyses by different digital strategies showed that instant message-based intervention was effective in improving ART adherence (RR = 2.40, 95% CI 1.39–4.17).

**Fig. 5:**
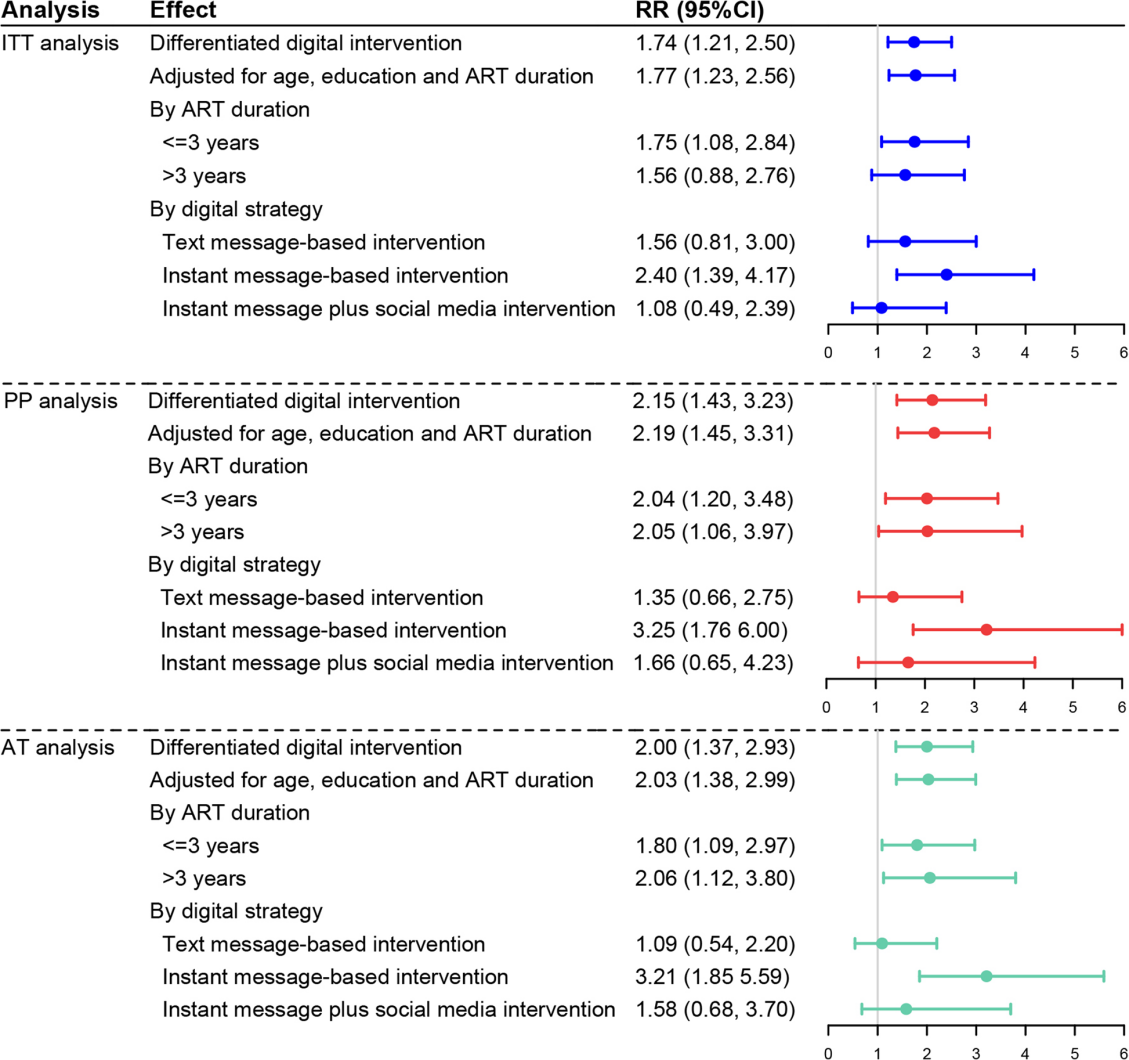
Effect of differentiated digital intervention on ART adherence among MSM living with HIV in China, 2020–2021. ART, antiretroviral therapy; RR, risk ratio; CI, confidence interval; ITT, intention-to-treat; PP, per-protocol; AT, as-treated

The PP and AT analysis produced similar results and the estimated effects were larger. In PP and AT analysis, the effect of differentiated digital intervention on ART adherence was 2.15 (95% CI 1.43–3.23) and 2.00 (95% CI 1.37–2.93) respectively. The effect of instant message-based intervention was 3.25 (95% CI 1.76–6.00) and 3.21 (95% CI 1.85–5.59), respectively. In addition, the differentiated digital intervention was effective among both those initiated ART ≤ 3 years (PP analysis: RR = 2.04, 95% CI 1.20–3.48; AT analysis: RR = 1.80, 95% CI 1.09–2.97) and >3 years (PP analysis: RR = 2.05, 95% CI 1.06–3.97; AT analysis: RR = 2.06, 95% CI 1.12–3.80) (Fig. 5). After combining the instant message subgroup and instant message plus social media subgroup, the intervention was still effective (ITT: RR = 1.81, 95% CI: 1.16–2.82; PP analysis: RR = 2.52, 95% CI 1.52–4.17; AT analysis: RR = 2.46, 95% CI 1.55–3.88) (Additional file 7: Figure S2). In addition, the absolute effect size (i.e., RD and 95% CI) for primary outcomes are presented in Table S1 of Additional file 8.

### Effect of differentiated digital intervention on secondary outcomes

Table 2 displays the results of secondary outcomes using ITT strategies. There was no significant difference in proportions of viral suppression (RR = 1.39, 95% CI 0.64–3.05), CD4 T cell counts (MD = − 1.55, 95% CI − 17.51–14.42), HIV treatment adherence self-efficacy (MD = 1.41, 95% CI − 0.09–2.92), and quality of life (MD = 0.47, 95% CI − 0.43–1.36). Subgroup analysis showed that instant message plus social media group intervention increased the psychological quality of life (MD = 0.64, 95% CI 0.16–1.11) (Additional file 9: Table S1). The sensitivity analysis (PP and AT) of secondary outcomes are displayed in Table S1-S2 of Additional file 10.

**Table 2:** Effect of differentiated digital intervention on secondary outcomes using ITT analysis among MSM living with HIV in China, 2020–2021

	Differentiated digital intervention (Total)	Text message-based intervention	Instant message-based intervention	Instant message plus social media group intervention
**Outcomes**	**RR (95%CI)**			
Viral suppression^a^	1.39 (0.64, 3.05)	1.28 (0.15, 10.59)	0.85 (0.27, 2.62)	3.06 (0.70, 13.33)
	**MD (95%CI)**			
CD4 T cell counts	− 1.55 (− 17.51, 14.42)	− 10.67 (− 39.38, 18.04)	6.98 (− 14.87, 28.84)	− 5.81 (− 42.58, 30.95)
HIV treatment adherence self-efficacy	1.41 (− 0.09, 2.92)	1.24 (− 1.55, 4.02)	1.67 (− 0.26, 3.61)	0.27 (− 3.45, 3.99)
Quality of life	0.47 (− 0.43, 1.36)	− 1.50 (− 3.24, 0.25)	0.85 (− 0.43, 2.13)	1.66 (− 0.16, 3.47)

*Abbreviations*: *ITT* intention-to-treat, *RR* risk ratio, *MD* mean difference, *CI* confidence interval, *MSM* men who have sex with men

^a^Defined as undetectable viral load (i.e., < 20 copies/ml)

## Discussion

ART adherence is critical to achieving viral suppression and reducing HIV-associated mortality and morbidity [40]. This study evaluated the effectiveness of a differentiated digital intervention on ART adherence among MSM living with HIV using a pragmatic RCT. We found that the differentiated digital intervention was effective in improving ART adherence. The one-to-one instant message was particularly useful in increasing ART adherence in terms of different digital strategies. This study expands the existing literature on differentiated digital interventions, selects digital media based on MSM preferences, and focuses on a middle-income country context [30]. Most digital HIV interventions focused on testing and were conducted in high-income countries [16].

Our data suggested that a differentiated digital intervention increased ART adherence among MSM living with HIV. Within our study cohort, 23.2% of participants (including 17.1% in the intervention arm and 28.9% in the control arm) self-reported suboptimal adherence at 6-months follow-up. The prevalence of suboptimal adherence was higher than that (4.8%) reported in an Asian cohort [41]. However, this cohort only measured percent adherence without considering dose-timing. Taking ART medications at irregular times can change its pharmacokinetic and pharmacodynamics effects, suggesting the equivalent importance of monitoring dose-timing adherence. Our finding contrasts with an RCT in China, which found that a combined digital intervention (text message and instant message) did not improve ART adherence (also be categorized as binary outcome based on ever missed doses within one-month period) [42]. One important difference was that all participants were allowed to decide the digital strategy based on their preferences in this study, suggesting that differentiation may enhance ART adherence [43]. Given that digital technologies offer the potential to reach large number of PLWH and differentiation strategy can improve engagement, similar differentiated digital interventions may be worth promoting in LMIC contexts.

Our findings showed that the differentiated intervention was effective among those who started ART more recently and long ago. Considering that patients with short ART duration were relatively inexperience, we speculated that the intervention may be particularly effective. However, PP and AT analyses showed that the intervention effect was larger among patients with longer ART duration. Previous cohort studies suggested that adherence would decrease with the extension of ART duration [44, 45]. In view of this, intensive and novel intervention may have greater room to promote adherence in this subgroup.

In this study, most MSM preferred one-to-one instant messaging and this digital strategy was effective. Our study utilized instant message platform to deliver multimedia (e.g., images and videos) materials, which volunteers designed from a MSM organization with extensive HIV intervention experience. Previous studies showed that community engagement and multimedia materials were more attractive and effective in delivering HIV interventions [46, 47]. In addition, we customized a WeChat mini-program as a reminder and clock-punching tool, and provided financial incentives for those who used online clock-punching. The WeChat mini-program attracted some participants’ engagement and assisted in developing habits of taking medications on time. About a quarter of MSM preferred text messaging, but it did not significantly improve ART adherence, which may be because simple text messages were not sufficient to sustain the attention of participants [48]. Contrary to expectations, the instant message plus social media intervention did not significantly improve ART adherence. Although we nominated the administrators to guide communications in the group, we could not prevent group members from contacting with each other in private. Unsupervised online communication could result in a number of unforeseen risks, including exposure to misleading information, facing hostile or derogatory comments from others, and feeling more uncertain about one’s health condition [49]. Additional research with larger samples is needed to further evaluate the group intervention effects.

After the intervention, we did not observe significant improvements in CD4 T cell counts and HIV viral suppression. It may be due to the relatively short duration of follow-up. Previous data showed that changes in biological outcomes could be delayed up to two years since reporting suboptimal ART adherence [50].

This study has several research and policy implications. First, the differentiated digital intervention is responsive to the needs of PLWH and can contribute to adherence and patient satisfaction. Policy makers can consider integrating this intervention model into PLWH management. Further, most participants preferred one-to-one instant messaging and the digital strategy was effective. Researchers should consider instant message delivery when planning HIV interventions among MSM living with HIV. Text messages may be complementary to expanding dissemination, especially for elders who cannot access the Internet and those who are more concerned about Internet privacy [51]. Finally, in addition to serving as an intervention component, WeChat mini-programs could also be a long-term adherence monitoring tool (tracking the records of clock-punching). The mini-program is more convenient, private, and cost-effective than the Wisepill device [52].

This study is subject to several limitations. First, the primary outcome of ART medication adherence was based on self-report. Nevertheless, previous studies showed that self-reported adherence was strongly associated with biological outcomes [53, 54]. We also collected biological outcomes (CD4 T cell counts and viral load) from medical records as secondary outcomes. Second, participants were allowed to choose digital strategy based on their preferences, and the end of recruitment was based on reaching the total sample size instead of satisfying the sample sizes of all three subgroups. Hence, the sample size of text message subgroup and instant message plus social media subgroup was slightly lower than expected, which may decrease power of the two subgroup analyses. Third, the generalizability of the study was limited because we only recruited participants from one site in China. These interventions did not generalize to other settings with fewer resources, varied access to technology and the Internet, and people with different cultural and social perspectives or lower literacy, and increased infection in the elderly. Further studies are needed among groups of different ages, cultures, and locations. In addition, there may be contamination between intervention and control arm. To evaluate the effect of contamination, we asked the participants whether they had seen materials that should only be delivered to intervention arm in the second follow-up, and then conducted PP and AT analyses. Fourth, adherence is a complex phenomenon and PLWH may become habituated to the intervention. The 6-month follow-up may be not enough to evaluate the long-term intervention effect considering that the effect of adherence interventions would wane over time [27]. A longer follow-up time would be important to understand sustainment over time.

## Conclusions

The differentiated digital intervention effectively improved ART adherence among MSM living with HIV in China. This intervention could be integrated into PLWH management and further considered in middle-income countries (e.g., Brazil, Nigeria) where there are large numbers of PLWH who can access text messaging or instant messaging services. In terms of different digital strategies, one-to-one instant message with multimedia technologies was particularly useful in increasing ART adherence. Instant message delivery may be considered as priority when planning HIV interventions among MSM living with HIV. The study contributed to recommendations to promote community engagement and multimedia technologies (e.g., images, videos, and mini program) in health service delivery and use differentiation strategy to expand service coverage.

## Supplementary Information

**Additional file 1.** Study protocol.

**Additional file 2.** CONSORT 2010 Checklist for reporting parallel group RCTs.

**Additional file 3. **Health messages and peer education stories. **Table S1**. ART medication messages. **Table S2**. HIV clinical messages. **Table S3**. Peer education stories. **Table S4**. Health behavior and nutrition messages.

**Additional file 4.** Short articles shared to QQ group.

**Additional file 5. **Basic characteristics of the participants in three digital subgroups. **Table S1**. Socio-demographic, behavioral and clinical characteristics of the study participants in three digital subgroups.

**Additional file 6. **Measurements of the secondary outcomes at baseline and two follow-ups. **Table S1**. Measurements of CD4 T cell counts and proportions of HIV viral suppression at baseline and two follow-ups. **Table S2**. Measurements of HIV treatment adherence self-efficacy at baseline and two follow-ups. **Table S3**. Measurements of quality of life at baseline and two follow-ups.

**Additional file 7. **Sensitivity analysis of combining two subgroups. **Figure S1**. Proportions of optimal ART adherence in the combined subgroups (i.e., instant message subgroup and instant message plus social media subgroup). **Figure S2**. Effect of the combined instant message-based and instant message plus social media intervention on ART adherence among MSM living with HIV in China, 2020-2021. Abbreviations: ART, antiretroviral therapy; RR, risk ratio; CI, confidence interval; ITT, intention-to-treat; PP, per-protocol; AT, as-treated.

**Additional file 8. **Absolute effect size of primary outcome. **Table S1**. Absolut effect size of differentiated digital intervention on ART adherence among MSM living with HIV in China, 2020-2021.

**Additional file 9. **Intervention effect on various domains of quality of life using ITT analysis. **Table S1**. Effect of differentiated digital intervention on various domains of quality of life using ITT analysis among MSM living with HIV in China, 2020-2021.

**Additional file 10. **Sensitivity analyses of the secondary outcomes. **Table S1**. Effect of differentiated digital intervention on secondary outcomes using PP analysis among MSM living with HIV in China, 2020-2021. **Table S2**. Effect of differentiated digital intervention on secondary outcomes using AT analysis among MSM living with HIV in China, 2020-2021.

## Data Availability

The datasets used and/or analyzed during the current study are available from the corresponding author (weima@sdu.edu.cn) on reasonable request.

## Abbreviations

ART: Antiretroviral therapy
AT: As-treated
CDC: Center for Disease Prevention and Control
CI: Confidence interval
GLMM: Generalized linear mixed model
ITT: Intention-to-treat
LMIC: Low- and middle-income country
MD: Mean difference
MSM: Men who have sex with men
PLWH: People living with HIV
PP: Per-protocol
RCT: Randomized controlled trial
RD: Risk difference
RR: Risk ratio
SMS: Short message service
STI: Sexually transmitted infection
UNAIDS: The Joint United Nations Programme on HIV/AIDS
USD: United States dollars
