# Supplementary material for: A differentiated digital intervention to improve antiretroviral therapy adherence among men who have sex with men living with HIV in China: a randomized controlled trial
Source: BMC Med. 2022 Oct 10;20:341. doi: 10.1186/s12916-022-02538-3 (PMC9549628; doi:10.1186/s12916-022-02538-3)
Supplement: Supplementary file 1 — Additional file 1. Study protocol. [file 12916_2022_2538_MOESM1_ESM.docx]

**Additional file 1**

**A differentiated digital intervention to improve antiretroviral therapy adherence of men who have sex with men (MSM) living with HIV in China: study protocol for a randomized controlled trial**

**1. List of abbreviations**

ART: Antiretroviral therapy

CDC: Centers for Disease Control and Prevention

GLMM: Generalized linear mixed model

HIV: Human Immunodeficiency Virus

HIV-ASES: HIV Treatment Adherence Self-Efficacy Scale

LMICs: Low-income and middle-income countries

MSM: Men who have sex with men

PLWH: People living with HIV

PI: primary investigator

RCT: Randomized controlled trial

SMS: Short message services

TPB: Theory of planned behavior

UI: User interface

WHO: World Health Organization

WHOQOL-HIV BREF: World Health Organization Quality of Life HIV short version

**2. Summary**

**Background:** The antiretroviral therapy (ART) requires a high level of adherence to ensure its effectiveness but adherence is suboptimal in many settings. Digital interventions have shown significant potential to improve ART adherence but there have been few ART interventions in China.

**Purpose:** This purpose of this study is to evaluate whether a differentiated digital intervention can improve ART adherence among men who have sex with men (MSM) living with HIV in China.

**Design:** This is a pragmatic randomized controlled trial (RCT) with two parallel arms including a baseline survey and two follow-ups with 3 months interval.

**Sites:** An infectious disease hospital (designated ART delivery site) in Jinan, Shandong Province of China

**Study populations and eligibility**: A total of 570 participants will be recruited. Eligibility criteria include MSM living with HIV who are currently on ART in the hospital, 18 years old or above, and willing to choose one of the three digital strategies (text message, only instant message or instant message plus social media group).

**Randomization**: Participants will be assigned to the intervention arm or the control arm through stratified block randomization (stratification=3, block sizes=4).

**Interventions**: The interventions include health messaging, medication reminders, peer education and online group discussion. Intervention contents are consistent but the ways of intervention delivery will be adapted based on different digital strategies.

**Study outcomes**: The primary outcome is ART adherence within the previous month. Secondary outcomes include CD4 T-cell counts, HIV detectable viral load, HIV treatment adherence self-efficacy and quality of life.

**Trial registration:** ChiCTR2000041282, retrospectively registered on 23 December 2020.

**Keywords:** Antiretroviral therapy adherence; Digital intervention; Social media; Men who have sex with men; Randomized controlled trial; China

**3. Introduction**

**3.1 Background**

Human Immunodeficiency Virus (HIV) infection remains a major public health contributor to disease burden worldwide, especially in low-income and middle-income countries (LMICs) [1, 2]. High Active Antiretroviral Therapy, introduced in mid-1990s, can effectively suppress HIV viral load and significantly reduce morbidity and mortality [3]. To ensure the effectiveness of antiretroviral therapy (ART), a high level of medication adherence (≥95%) is required [4]. However, strict adherence to a precise HIV treatment regimen has not been achieved yet in many regions. For example, a recent meta-analysis in China found that the mean rate of ≥95% adherence to ART was 81.1% at one week and dropped to 68.3% at three months or longer [5]. To achieve full adherence to avoid treatment failure, innovative approaches are needed.

Digital interventions are defined as interventions using digital technologies such as smartphones, website, and text messaging [6]. In the past decade, digital interventions have emerged as a promising solution to improve ART adherence. Particularly, short message services (SMS) interventions have been widely used due to convenience and cost-effectiveness [7-11], and are recommended by the World Health Organization (WHO) [12]. Further, social media have been used to deliver HIV interventions, especially for marginalized populations. Social media is defined as Internet-based platforms allowing the creation and exchange of user-generated contents, which usually applies mobile or Web-based technologies [13]. Studies have shown that social media can be used to promote ART adherence among key populations [14-16]. Social media offers the potential for public health practitioners to reach a larger number of people living with HIV (PLWH) and to disseminate information on sensitive topics [17]. However, most studies that focus on social media interventions were conducted in high-income countries [17]. In addition, there are few studies that have allowed people to decide which intervention they want to use, despite the known importance of preferences and people-centered approaches.

In China, there were 1,045,000 reported cases of PLWH by the end of October 2020 [18]. Among them, men who have sex with men (MSM) accounted for 23% of newly-diagnosed cases in 2019 [19]. WeChat and Tencent QQ are two of the most popular Chinese social media platform, especially for youngsters. As of 2016, WeChat had connected more than half a billion Chinese people and QQ had over 700 million active users [20, 21]. In China, there have been studies adopting social media to deliver interventions but most focused on HIV testing [13, 22, 23]. To our knowledge, only one study in South China explored the feasibility of WeChat-based intervention for ART adherence but did not observe significant improvements [24]. Another smartphone APP-based study was a protocol [25]. Few studies using digital interventions to promote ART adherence have been tested with rigorous design despite the high rates of mobile phone use [26, 27]. Therefore, we developed a differentiated comprehensive intervention which allows patients to choose different digital strategies (text message, instant message, or instant message plus social media group) based on their preferences. The differentiated intervention indicates that the ways of intervention delivery are differentiated among the subgroups.

**3.2 Objectives**

**Primary objective**: To determine if the differentiated digital intervention can improve ART adherence among MSM living with HIV at 6 months compared to the control arm.

**Hypotheses 1**: The differentiated digital intervention is superior in improving ART adherence compared to the control arm.

**Secondary objectives**: To determine (1) whether CD4 T-cell counts, HIV viral suppression, HIV treatment adherence self-efficacy and quality of life differ between the intervention arm and the control arm at 6 months; (2) whether the outcomes (i.e. ART adherence, CD4 T-cell counts, HIV viral suppression, HIV treatment self-efficacy and quality of life) differ between the two study arms within each of the three subgroups (i.e. text message subgroup, instant message subgroup, and instant message plus social media subgroup)

**Hypotheses 2**: The differentiated digital intervention is superior in improving CD4 T-cell counts, HIV viral suppression, HIV treatment adherence self-efficacy and quality of life. Text message-based intervention, instant message-based intervention, and instant message plus social media intervention are superior in improving the health outcomes.

**4. Intervention development** **and** **theoretical support**

The development of intervention was guided by the theory of planned behavior (TPB) [28] and group medical visit (or shared medical appointments) [29]. According to TPB, the most important determinant of behavior is behavioral intention. The direct determinant of one’s behavioral intention include attitude toward performing the behavior, subjective norm associated with the behavior, and perceived control over the behavior [28]. The TPB has been widely applied in sex behavior, condom use, HIV testing, and ART adherence among MSM [30-33]. Specifically, based on TPB, we plan to disseminate health messages on ART medication to strengthen behavioral beliefs about outcomes of performing the behavior, and contribute to form positive attitudes toward taking medications. Further, we will provide peer education to improve subject norm of ART adherence. An individual who believes important referents (peer educators) think he should perform a behavior and is motivated to meet expectations of the referents tends to hold positive subjective norm. Simultaneously, we will provide medication reminders and use incentives to strengthen individuals’ control belief and perceived power, and further improve perceived behavioral control. Besides, we plan to establish an online chat group to encourage discussions about ART experience based on group medical visit. The model framework of the intervention is displayed in Figure S1.

We conducted a scoping literature review on digital technologies and adherence intervention strategies. In terms of digital technologies, we found that most HIV digital interventions focused on testing and were conducted in high-income countries [13]. In terms of intervention strategies on ART adherence, we found that interventions recommended by WHO guidelines include peer counsellors, mobile phone text messages, reminder devices, and behavioral skills training et al [12]. In addition, literature showed multiple strategies were superior to a single strategy [34].

Prior to launching the study, we conducted formative research targeting MSM living with HIV in Yantai and Weifang of Shandong Province, China. We focused on the following themes: whether they had any difficulties or needs on ART at present, whether they would like to receive health services via digital platforms (and which digital platforms they would like to choose), whether they would like to join an online chat group to share ART experience with other patients (and the reasons if not). Simultaneously, we asked the health providers from local hospital and CDC about the feasibility and acceptability of implementing digital interventions among MSM living with HIV.

The final interventions include health messaging, medication reminders, peer education, and online group discussion. We will deliver interventions using different digital strategies (including text message, only instant message, and instant message plus social media group) based on patients’ preferences.

**Attitude**

Disseminating health messages about ART medication to improve attitudes to ART

**Subject norm**

Providing peer education to improve subject norm of ART adherence

**Perceived behavioral control**

Providing medication reminders and incentives to strengthen perceived behavioral control

**Group visit (inter-person level)**

Establishing an online chat group to encourage discussions about ART experience

**Behavior Intention**

Improving the behavior intention of taking ART medications

**Behavior**

Keeping consistent optimal ART adherence

TPB (individual level)

Patients’ need and preferences

Differentiated digital intervention

**Figure S1. Model framework of differentiated digital intervention**

**5. Overview of design**

This study will be a pragmatic RCT with two parallel arms designed for MSM living with HIV currently on ART. Participants will be randomly assigned in a 1:1 ratio to the intervention arm or the control arm, stratified by different digital strategies. For each stratum, block randomization will be triggered once four participants are recruited. Participants in the intervention arm will receive 6-month comprehensive interventions including ART medication messages, medication reminders, peer education and online group discussion. Participants in the control arm will receive messages on health behavior and nutrition. The study will last about 7.5 months. The first 1.5 months include the recruitment period and the baseline survey. Two follow-up surveys will be administrated every 3 months.

The flowchart of this differentiated digital intervention trial is displayed in Figure S2. The SPIRIT figure of study timeline is displayed in Figure S3.

**Figure S2. Flowchart of the differentiated digital intervention trial.**

**
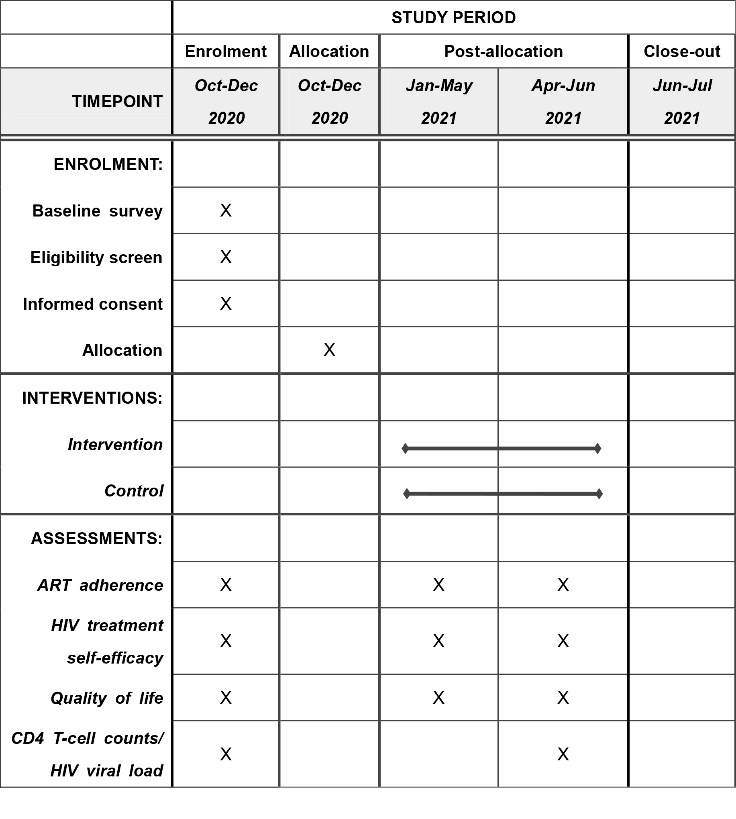
**

**Figure S3. SPIRIT figure of study timeline**

**6. Trial population and study settting**

**6.1 Eligibility**

The participation eligibility criteria are: (1) HIV-diagnosed seropositive and currently on ART in the local hospital of study setting; (2) reaching 18 years old or above; (3) born biologically male and having ever had anal sex with men; (4) willing to choose one of the three provided digital strategy (text message, instant message or instant message plus social media group) and provide phone number or WeChat account (plus QQ account).

**6.2 Study setting**

The participants will be recruited from an infectious diseases hospital that serves more than 1,600 MSM patients in Jinan, Shandong Province of China.

**6.3 Recruitment**

Patients waiting to take free antiretroviral medications will be invited to a waiting room through physician referral and receive a detailed introduction of this study by trained research staff. If a patient meets the eligibility criteria, he will be asked to provide written informed consent and complete the baseline questionnaire. The treatment ID of each participant will be collected so that they can be identified in the follow-ups. In addition, based on the digital strategies they choose, participants need to provide their phone number or scan QR codes to add a WeChat account of the project as contact. For those choosing WeChat+QQ service, we will first add them as WeChat contacts, and then share QR codes to invite them to join in QQ group for some participants. The participants will be reimbursed about 7.47 USD for each of the survey questionnaire completed (including a baseline survey and two follow-up surveys).

**7. Randomization and blinding**

When the participants have completed the baseline survey, they will be randomly assigned in a 1:1 ratio to the intervention arm or the control arm. The procedure of randomization will be stratified according to participants’ preferred digital strategy (n=3). Allocation will be conducted by a computer-generated randomization list with block sizes of four within each stratum using SAS software version 9.4 (SAS Institute, Inc., Cary, NC, USA).

A research assistant (CR) with no involvement in the trial will generate the computerized random allocation sequence. CR will also perform the packaging of sequentially numbered, opaque, sealed envelopes. The health providers (including CW, GL, YB, XZ, and QC) and investigators (including YY, CC, YL, WJ, LW, YC, ZZ, XY, and JM) will participate in enrolling participants. They will be masked to random allocation sequence during the trial. The investigators will assign participants to interventions. Allocation concealment will be ensured, as the envelopes will not be opened before the participant has been irreversibly included in the study.

Due to the nature of the design, participants will not be blinded to their way of receiving service. The intervention providers will be aware of study assignment. But all field surveys and analyses will be conducted by team members who are blinded to the assignments of intervention arm and control arm.

**8. Interventions**

Participants in the intervention arm will receive interventions within six months post allocation, which include health messaging, medication reminders, peer education and online group discussion. The health messages include 12-item ART medication messages and 6-item HIV clinical messages, which are adopted from national AIDS antiviral treatment manual or authoritative websites of China’s CDC, and checked by AIDS experts from local hospital and CDCs. The 12-item ART medication messages include management of side effects, solutions for missing doses, definition of drug resistance, etc. The 6-item HIV clinical messages include explanations of some HIV terms (e.g. “window period of HIV infection”, “CD4 T-cell counts”, “HIV viral load”), precondition of ART initiation, etc. We will deliver ART medication messages every two weeks and HIV clinical messages every month respectively. Second, daily medication reminder service (SMS platform or WeChat mini-program) is designed to serve for those who need the service (optional for participants). The reminders will not include words regarding “HIV” or “AIDS” to avoid potential privacy disclosures. Third, peer education stories will be used to improve ART adherence and health perception. We will deliver peer education service every two weeks after the first follow-up (i.e. 3 months post allocation). The ways of intervention delivery are differentiated for each subgroup, the details of which are as followed.

**8.1 Text message-based intervention**

Participants will receive ART medication messages and HIV clinical messages via SMS. Besides, participants will be asked if they need daily medication reminders in the first intervention. If the response is “yes”, the daily reminders “Have you finished the task today? Remember to keep in a good mood everyday” will be sent to them about three minutes before their prescribed dose-timing via a customized SMS platform. In addition, peer education stories will be delivered to the participants via SMS after the first follow-up.

**8.2 Instant message-based intervention**

A total of 18 images of ART medication messages (n=12) and HIV clinical messages (n=6) plus six peer education videos will be delivered to participants via WeChat. The images are designed by peer volunteers from a community-based MSM organization to better attract participants (see four examples in Figure S4). In addition, we also invite peer volunteers living with HIV from a MSM organization to record six peer education videos to promote ART adherence and health perception.


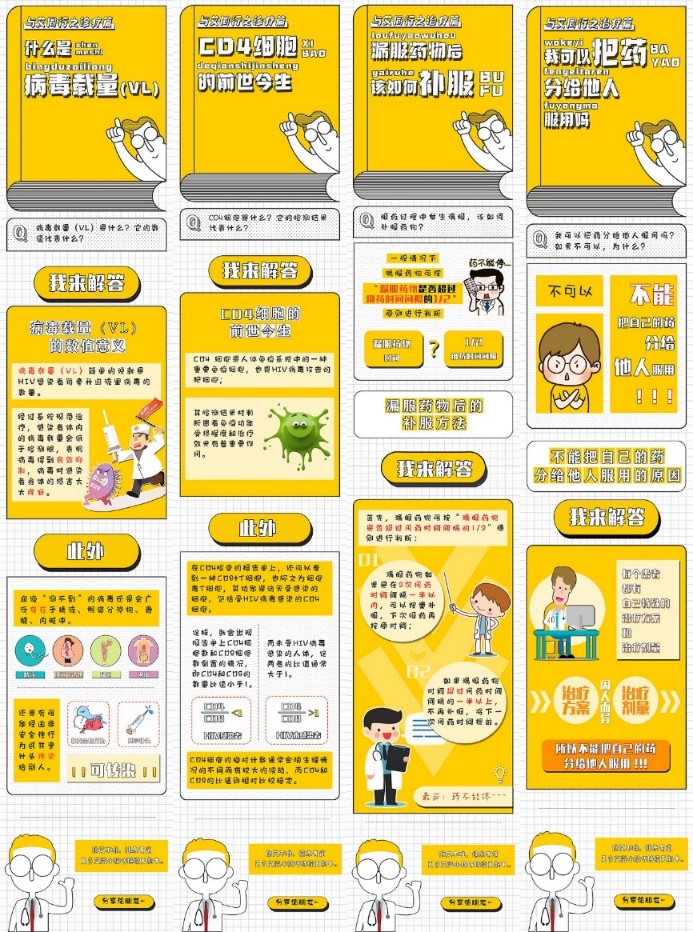


**Figure S4 Four of the eighteen images of HIV clinical related knowledge and ART medication knowledge used in the intervention package**

Note: The themes of the four images from left to right are: (1) What is HIV viral load; (2) What is CD4 T-cell counts; (3) How to complement ART medications if missing doses; (4) Whether it is okay to share your own ART medications to others. Example (Left text): “What is the HIV viral load: Viral load (VL) is the number of viruses per milliliter of blood. After standardized treatment, VL will be lower than the detection limit, indicating that the virus has been effectively suppressed and the damage of the virus to body has been greatly reduced. However, undetectable viruses in blood still distribute in semen, vaginal secretions, bone marrow and internal organs, and may be transmitted to others through unsafe sex or sharing needles.”

A customized WeChat mini-program entitled “U=U” (Undetectable=Untransmittable) is developed to remind participants to take medications on time and also provide them the opportunity of online clock-punching after taking medication. WeChat mini-program, a revolutionary lightweight “APP” without the need of downloading, can significantly improve accessibility and convenience [35]. First, the research staff will send QR codes and manual of the mini-program to participants via WeChat. Those who are interested in this service can scan QR codes to authorize the login of their WeChat accounts. After that, research staff can identify them from the management platform of this mini-program and type in their ART medication information (i.e. prescribed medication time and frequency of daily medication-taking) collected from baseline questionnaire. Then the participants can enter the user interface (UI) of the mini-program (Figure S5) to complete online clock-punching. The valid time of clock-punching is limited to one hour after prescribed medication time. If the participants try to punch the clock beyond the valid time, a prompt window “Sorry, invalid clock-punching for timeout” will pop up. The participants will be awarded about 4.48 USD if they complete 30-days uninterrupted clock-punching and the reward is cumulative. A clock-punching calendar recording each participant’s own valid clock-punching dates is also available in UI of the WeChat mini-program (Figure 4). Also, participants who need daily medication reminders can receive the service delivered by this mini-program three minutes ahead of their prescribed dose-timing.


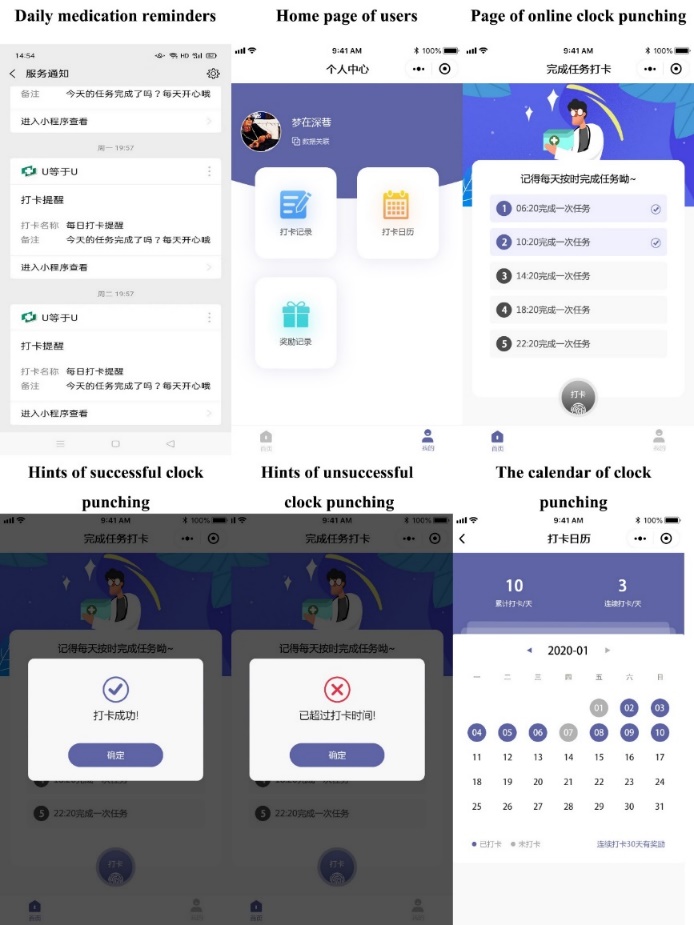


**Figure S5 The users’ interfaces (UI) of the WeChat mini program**

**8.3 Instant message plus social media group intervention**

Based on instant message-based intervention, a social media group will be established to explore the effectiveness of inter-person intervention. QQ group is one of the main functions of QQ allowing multiple users to communicate instantly. The QQ group can only be found through ID or name. Participants need to scan the QR codes shared by a team member and to receive permission of the group administrator to join the group. A team member (the group creator and administrator) and a STD physician from the hospital will be invited into this QQ group to facilitate mutual medication reminding and discussion about ART experience among participants. In addition, short articles about frontier HIV research from reliable sources (hospital, CDCs, CBOs, etc.) will be shared to this group every week.

**8.4 Control**

For participants in the control arm, 12-item HIV health behavioral messages tailored for MSM and nutrition messages targeted HIV/AIDS patients will be delivered every two weeks via SMS or WeChat based on their choices. All of these messages are adopted from National Center for AIDS/STD Control and Prevention and National Institute for Nutrition and Health.

**9. Outcomes and measures**

**9.1 Primary outcome**

The primary outcome is proportion of ART medication adherence within the previous month, which will be evaluated at baseline and in each follow-up. We will take both the percent adherence and dose-timing adherence into account to get a comprehensive assessment of ART adherence.

The ART adherence will be measured by two questions including “How many times have you missed taking your antiretroviral medication in the past month” and “how often did you take medications within one hour of prescribed dose-timing in the past month”. The first question requires participants to fill in the counts of missed doses in the past month. Options for the second question will include “never”, “occasionally”, “nearly half the time”, “more than half the time (most of the time)” and “all within the prescribed time”. We will categorize ART adherence as a binary variable. Those who never miss any doses or delay doses by one hour within the past month will be defined as “optimal adherence”. Otherwise, individuals will be regarded as “suboptimal adherence”.

**9.2 Secondary outcomes**

The secondary outcomes include CD4 T-cell counts, HIV viral suppression, HIV treatment adherence self-efficacy and quality of life. Among them, CD4 T-cell counts and HIV viral load will be obtained from medical records. The designated ART hospital regularly provides free CD4 T-cells and HIV viral load testing for all patients from March to June every year, the time of which is basically consistent with our planned second follow-up. In view of this, we will collect data in 2020 as baseline (pre-intervention) measurement and data in 2021 as post-intervention measurement. We will not collect additionally biological samples from participants in this study.

HIV treatment adherence self-efficacy will be measured using HIV Treatment Adherence Self-Efficacy Scale (HIV-ASES) at baseline and in each follow-up. The HIV-ASES was released by American CDC [36] and revised to Chinese version by Sun et al. [37]. The revised HIV-ASES-Chinese-Version includes 12 items measuring 2 dimensions (integration and perseverance). Respondents will rate on an 11-point Likert scale about how confident they are at performing behaviors related ART treatment (from 0= “not at all confident” to 10= “totally confident”). Scores of HIV-ASES range from 0 to 120, with higher score indicating better levels of self-efficacy. The revised HIV-ASES displays high reliability and validity (Cronbach’s alpha=0.876, 95%CI: 0.868-0.885) [37].

Quality of life will be evaluated by World Health Organization Quality of Life HIV short version (WHOQOL-HIV BREF) [38] at baseline and in each follow-up, which consists of 31 items covering 6 domains (physical, psychological, level of independence, social relationships, environment, and beliefs). The scores of WHOQOL-HIV BREF can be calculated within each domain or in total, ranging from 4 to 20 and 24 to 100 respectively, with higher score representing a higher level of quality of life.

**10. Sample size calculation**

The sample size was guided by a Gunn et al. [39]. The primary objective is to compare ART medication adherence at 6 months post allocation between the intervention arm and the control arm. The calculation was based on planned subgroup analysis, for that sample size would need to be larger to observe differences between study arms within strata than combined. Based on a previous SMS intervention in China [26], we calculated the sample size to detect a mean difference of 0.15 in proportions of achieving optimal ART adherence for text message subgroup. Due to no preliminary data, we hypothesized the difference will be no less than 0.15 for the instant message subgroup and instant message plus social media subgroup because participants in these two groups will receive consistent interventions and more incentives (cumulative clock-punching incentive), and consequently may have larger potential for adherence improvement.

Therefore, assuming a mean difference of 0.15 in proportions of achieving optimal adherence, two-sided alpha=0.05, 80% power and 20% loss to follow up, the sample size is 190 for text message subgroup. According to our pilot survey in another infectious disease hospital of Shandong Province, about two-thirds of PLWH are willing to choose instant message, and the others use text message or phone. Consequently, we anticipated that one-third of participants would choose text message in this study and extrapolated the total sample size was 570. The sample size calculation is conducted by PASS 15.

Due to the nature of the design, we cannot ensure participants choosing each of the three digital strategies in the anticipated proportion. And due to the limitation of cost and timeline, we will end up recruiting participants until reaching the total sample size (n=570) instead of satisfying the sample sizes of all three subgroups (each group n=190).

**11. Data collection**

Data will be collected using written questionnaires. At baseline, we will collect information about socio-demographic characteristics (including age, household, education, occupation, monthly income and marital status, etc), behavioral characteristics (including homosexual behaviors, sexual orientation disclosure, HIV status disclosure), HIV clinical knowledge, basic information of ART (including time and frequency of daily medication-taking, frequency of missed doses in the month, reasons of missed doses, side effects, etc), HIV treatment adherence self-efficacy, ART medication knowledge and quality of life. In follow-ups, we will collect information about ART adherence within the past month, HIV clinical knowledge, ART medication knowledge, HIV treatment adherence self-efficacy and quality of life through self-administered questionnaire. Besides, we will collect CD4 T-cell counts and HIV detectable viral load from medical records in the hospital.

A verification mechanism has been adopted to improve data quality. When a questionnaire is completed, investigators will check immediately to identify logical errors or missing data to verify with the participants. Participants will be reimbursed after the verification is completed.

**12. Analysis plan**

**12.1 Descriptive analysis**

All survey questionnaires will be coded and entered in to EpiData version 3.1 (EpiData Association, Odense, Denmark). Descriptive statistics will be used to compare the participants’ basic characteristics between the intervention and the control arm both in total and in each stratum. Student’s t tests will be used for normally-distributed continuous variables, Mann-Whitney U tests will be used for non-normally-distributed continuous variables, and Chi-square tests or Fisher’s exact probabilities will be used for categorical variables.

**12.2 Primary analysis**

The primary analysis will be performed on an intention-to-treat analysis utilizing generalized linear mixed models (GLMMs) to evaluate the effect of the differentiated digital intervention on the primary and secondary outcomes, which will include intervention status as fixed effects and individual participants with multiple measurements as random effects. The GLMMs will adjust for the baseline outcome measures (where appropriate), stratification factors (i.e. text message, instant message, instant message plus social media group), and time (baseline, 3 months and 6 months). Baseline variables significantly associated with the outcomes and found to be imbalanced between intervention arm and control arm will also be considered for adjustment in the regression models. The risk ratio (RR) or mean difference (MD) of the outcomes between study arms will be reported as estimated intervention effects with 95% confidence intervals and two-sided P values.

**12.3 Sensitivity analysis**

In addition, per-protocol analysis and as-treated analysis with GLMMs will be performed to assess the effect of contamination.

**12.4 Missing value**

For potential missing data, GLMM allow data from completed survey to be retained for analysis. A complete-case analysis will be used if an outcome variable miss for <15% of participants. Otherwise, sensitivity analysis using multiple imputations will be conducted to assess the robustness of the missing data.

**12.5 Subgroup analysis**

Subgroup analysis by different digital strategies will be carried out to compare the primary and secondary outcomes between the study arms within each of the three strata. As we mentioned before, the sample size may not be sufficient for all three subgroups. But based on the preliminary data and sample size calculation, we can ensure sufficient power in total and at least one of the three intervention subgroups.

**13. Ethical considerations**

**13.1 Adverse events and benefits**

**13.1.1 Potential adverse events**

Participants may feel embarrassed or anxious when we collect some privacy information. Participants may fear of privacy disclosure when we deliver interventions using SMS or WeChat. We will deliver interventions during non-work hours and adapt it based on participants’ requirement. We will capture potential adverse events via spontaneous self-report. Participants can contact us through phone or WeChat to report adverse events. If any participant feel they have experienced an adverse event from participating in this trial, they can withdraw at any time.

**13.1.2 Benefits of this study**

The comprehensive intervention of this study may promote participants to obtain health messages about ART, develop good habits of taking medications on time, get psychological and social support, and eventually improve ART adherence and quality of life. In addition, some participants may receive financial incentives by participating in our online clock-punching activities.

This study will provide reference for digital intervention (especially for Internet-based digital intervention) on ART adherence in Chinese contexts. Once proven effective, this intervention can be integrated into PLWH management and further considered in LMICs where there are large numbers of PLWH who can access text messaging or instant messaging services.

**13.2 Protocol amendments**

If any protocol amendments are needed over the course of the trial, these changes will be reported to relevant parties by email. Results of this trial will be distributed to national stakeholders.

**13.3 Monitoring**

An external data monitoring committee will not be formed for this study because the potential harm to participants is minimal. But we will establish an internal expert advisory committee (including university professors, clinical practitioners, CDC staff, and leaders of MSM organization) to guide protocol formation, data collection, data management, and data analysis. Regular meetings will be established to review trial progress. All participants are informed that they can withdraw at any time and they are provided a phone number or WeChat account to contact the primary investigator (PI) with any questions or concerns.

**13.4 Compliance**

Dr. Wei Ma and Dr. Dianmin Kang will lead the development and implementation of the differentiated digital intervention and overall study coordination. Dr. Meizhen Liao will be responsible for fiscal administration. The PIs will communicate regularly by phone or email to discuss study design, trial progress, and potential problems.

**13.5 Confidentiality**

All written questionnaires and informed consents were stored in locked, fireproof file cabinets. Meanwhile, all survey questionnaires will be entered into a secure computer with passwords and firewalls. This computer will be used for saving survey data. A smartphone with passwords will be used to save participants’ personal information (including phone number, WeChat account and QQ account) and deliver digital interventions for all participants. Only a PI and a designated health provider from the study site will have the password to access the smartphone.

**13.6 Ethical approval**

This study was reviewed and approved by the Ethical Review Committee of School of Public Health in Shandong University (20190210).

**13.7 Informed consent**

Before participating the baseline survey, all participants will be presented with a written informed consent. Once participants provide written consent (pseudonymous is allowed), they are required to complete study questionnaire.

**14. References**

1. Maartens G, Celum C, Lewin SR. HIV infection: epidemiology, pathogenesis, treatment, and prevention. Lancet. 2014; 384(9939):258-271.

2. UNAIDS. Fact sheet - latest statistics on the status of the AIDS epidemic. 2020. http://www.unaids.org/en/resources/fact-sheet. Accessed March 15 2021.

3. Bhaskaran K, Hamouda O, Sannes M, Boufassa F, Johnson AM, Lambert PC, et al. Changes in the risk of death after HIV seroconversion compared with mortality in the general population. JAMA. 2008; 300(1):51-59.

4. Paterson DL, Swindells S, Mohr J, Brester M, Vergis EN, Squier C, et al. Adherence to protease inhibitor therapy and outcomes in patients with HIV infection. Ann Intern Med. 2000; 133(1):21-30.

5. Wang YY, Jin Y, Chen C, Zheng W, Wang SB, Ungvari GS, at al. Meta-analysis of adherence to highly active antiretroviral therapy in patients with HIV infection in China. AIDS Care. 2019; 31(8):913-922.

6. Murray E, Hekler EB, Andersson G, Collins LM, Doherty A, Hollis C, et al. Evaluating Digital Health Interventions: Key Questions and Approaches. Am J Prev Med. 2016; 51(5):843-851.

7. Lester RT, Ritvo P, Mills EJ, Kariri A, Karanja S, Chung MH, et al. Effects of a mobile phone short message service on antiretroviral treatment adherence in Kenya (WelTel Kenya1): a randomised trial. Lancet. 2010; 376(9755):1838-1845.

8. Horvath T, Azman H, Kennedy GE, Rutherford GW. Mobile phone text messaging for promoting adherence to antiretroviral therapy in patients with HIV infection. Cochrane Database Syst Rev. 2012; 2012(3):CD009756.

9. Pop-Eleches C, Thirumurthy H, Habyarimana JP, Zivin JG, Goldstein MP, de Walque D, et al. Mobile phone technologies improve adherence to antiretroviral treatment in a resource-limited setting: a randomized controlled trial of text message reminders. AIDS. 2011; 25(6):825-834.

10. Kalichman SC, Kalichman MO, Cherry C, Eaton LA, Cruess D, Schinazi RF. Randomized Factorial Trial of Phone-Delivered Support Counseling and Daily Text Message Reminders for HIV Treatment Adherence. J Acquir Immune Defic Syndr. 2016; 73(1):47-54.

11. Finitsis DJ, Pellowski JA, Johnson BT. Text message intervention designs to promote adherence to antiretroviral therapy (ART): a meta-analysis of randomized controlled trials. PLoS One. 2014; 9(2):e88166.

12. World Health Organization. Consolidated Guidelines on the Use of Antiretroviral Drugs for Treating and Preventing HIV Infection 2016 Recommendations for a public health approach. Switzerland; 2016. http://www.who.int/hiv/pub/arv/arv-2016/en/. Accessed March 15 2021.

13. Cao B, Gupta S, Wang J, Hightow-Weidman LB, Muessig KE, Tang W, et al. Social Media Interventions to Promote HIV Testing, Linkage, Adherence, and Retention: Systematic Review and Meta-Analysis. J Med Internet Res. 2017; 19(11):e394.

14. Muessig KE, LeGrand S, Horvath KJ, Bauermeister JA, Hightow-Weidman LB. Recent mobile health interventions to support medication adherence among HIV-positive MSM. Curr Opin HIV AIDS. 2017; 12(5):432-441.

15. Horvath KJ, Alemu D, Danh T, Baker JV, Carrico AW. Creating Effective Mobile Phone Apps to Optimize Antiretroviral Therapy Adherence: Perspectives From Stimulant-Using HIV-Positive Men Who Have Sex With Men. JMIR Mhealth Uhealth. 2016; 4(2):e48.

16. LeGrand S, Muessig KE, McNulty T, Soni K, Knudtson K, Lemann A, et al. Epic Allies: Development of a Gaming App to Improve Antiretroviral Therapy Adherence Among Young HIV-Positive Men Who Have Sex With Men. JMIR Serious Games. 2016; 4(1):e6.

17. Garett R, Smith J, Young SD. A Review of Social Media Technologies Across the Global HIV Care Continuum. Curr Opin Psychol. 2016; 9:56-66.

18. Chuai Z, Zhang Y, Zhao Y, Yan J, Sun Z, Wang Y, et al. Latest AIDS epidemic in global and China. Infectious Disease Information. 2020; 33(06):501-503. (In Chinese)

19. Bureau of Disease Control and Prevention. The new progress in AIDS control and prevention in China in 2019. Chinese Journal of AIDS & STD. 2019; 25(12):1205. (In Chinese)

20. Zeng F, Deng G, Wang Z, Liu L. WebChat: a new clinical teaching tool for problem-based learning. International Journal of Medical Education. 2016; 7:119-121.

21. You ZQ, Han XP, Lu L, Yeung CH. Empirical Studies on the Network of Social Groups: The Case of Tencent QQ. PLoS One. 2015; 10(7):e130538.

22. Tang W, Wei C, Cao B, Wu D, Li KT, Lu H, et al. Crowdsourcing to expand HIV testing among men who have sex with men in China: A closed cohort stepped wedge cluster randomized controlled trial. PLoS Med. 2018; 15(8):e1002645.

23. Cao B, Liu C, Durvasula M, Tang W, Pan S, Saffer AJ, et al. Social Media Engagement and HIV Testing Among Men Who Have Sex With Men in China: A Nationwide Cross-Sectional Survey. J Med Internet Res. 2017; 19(7):e251.

24. Guo Y, Xu Z, Qiao J, Hong YA, Zhang H, Zeng C, et al. Development and Feasibility Testing of an mHealth (Text Message and WeChat) Intervention to Improve the Medication Adherence and Quality of Life of People Living with HIV in China: Pilot Randomized Controlled Trial. JMIR Mhealth Uhealth. 2018; 6(9):e10274.

25. Fan X, She R, Liu C, Zhong H, Lau J, Hao C, et al: Evaluation of smartphone APP-based case-management services among antiretroviral treatment-naive HIV-positive men who have sex with men: a randomized controlled trial protocol. BMC Public Health. 2020; 20(1):85.

26. Ruan Y, Xiao X, Chen J, Li X, Williams AB, Wang H. Acceptability and efficacy of interactive short message service intervention in improving HIV medication adherence in Chinese antiretroviral treatment-naive individuals. Patient Prefer Adherence. 2017; 11:221-228.

27. Sabin LL, Bachman DeSilva M, Gill CJ, Zhong L, Vian T, Xie W, et al. Improving Adherence to Antiretroviral Therapy With Triggered Real-time Text Message Reminders. J Acquir Immune Defic Syndr. 2015; 69(5):551-559.

28. AJZEN I. The Theory of Planned Behavior. ORGAN BEHAV HUM DEC 1991, 50(2):179-211.

29. Thompson-Lastad A. Group medical visits as participatory care in community health centers. Qual Health Res. 2018; 28(7):1065-76.

30. Andrew BJ, Mullan BA, de Wit JB, Monds LA, Todd J, et al. Does the Theory of Planned Behaviour Explain Condom Use Behaviour Among Men Who have Sex with Men? A Meta-analytic Review of the Literature. AIDS Behav. 2016;20: 2834-2844.

31. Thoma BC, Huebner DM. Parent-Adolescent Communication About Sex and Condom Use Among Young Men Who Have Sex With Men: An Examination of the Theory of Planned Behavior. Ann Behav Med. 2018;52: 973-987.

32. Chu JH, Huang JH. A Theory-Based Exploration of Condomless Anal Intercourse Intention Among Young Men Who Have Sex with Men of Different Sexual Roles in Taiwan. Arch Sex Behav. 2018;47: 2041-2050.

33. Sheehan DM, De La Cruz Y, Ramirez-Ortiz D, Swendeman D, Munoz-Laboy M, et al. A qualitative study of antiretroviral therapy adherence interruptions among young Latino men who have sex with men with HIV: Project D.A.I.L.Y. AIDS Care. 2021;33: 962-969.

34. Kanters S, Park JJ, Chan K, Socias ME, Ford N, Forrest JI, et al. Interventions to improve adherence to antiretroviral therapy: A systematic review and network meta-analysis. Lancet HIV. 2017; 4(1):e31-40.

35. Hao L, Wan F, Ma N, Wang Y. Analysis of the Development of WeChat Mini Program. Journal of Physics: Conference Series. 2018; 1087:62040.

36. Johnson MO, Neilands TB, Dilworth SE, Morin SF, Remien RH, Chesney MA. The role of self-efficacy in HIV treatment adherence: validation of the HIV Treatment Adherence Self-Efficacy Scale (HIV-ASES). J Behav Med. 2007; 30(5):359-370.

37. Sun L, Yang SM, Wu H, Chen B, Wang CJ, Li XF. Reliability and validity of the Chinese version of the HIV Treatment Adherence Self-Efficacy Scale in mainland China. Int J STD AIDS. 2017; 28(8):829-837.

38. O'Connell KA, Skevington SM. An international quality of life instrument to assess wellbeing in adults who are HIV-positive: a short form of the WHOQOL-HIV (31 items). AIDS Behav. 2012; 16(2):452-460.

39. Gunn J, Wachtler C, Fletcher S, Davidson S, Mihalopoulos C, Palmer V, et al. Target-D: a stratified individually randomized controlled trial of the diamond clinical prediction tool to triage and target treatment for depressive symptoms in general practice: study protocol for a randomized controlled trial. Trials. 2017; 18(1).
