# Supplementary material for: A differentiated digital intervention to improve antiretroviral therapy adherence among men who have sex with men living with HIV in China: a randomized controlled trial
Source: BMC Med. 2022 Oct 10;20:341. doi: 10.1186/s12916-022-02538-3 (PMC9549628; doi:10.1186/s12916-022-02538-3)
Supplement: Supplementary file 3 — Additional file 3. Health messages and peer education stories. Table S1. ART medication messages. Table S2. HIV clinical messages. Table S3. Peer education stories. Table S4. Health behavior and nutrition messages. [file 12916_2022_2538_MOESM3_ESM.docx]

**Additional file 3**

**Health messages and peer education stories**

**Table S1. ART medication messages**

| **Item** | **ART medication messages (delivered to intervention arm)** |
| --- | --- |
| 1 | Though ART cannot cure AIDS, it can inhibit viral replication, prolong patients’ life and improve the quality of life. |
| 2 | In general, missing doses can be judged according to the principle of "whether the time of missing doses overruns 1/2 of the prescribed dose-timing interval". If the missing doses occurs within half of the prescribed dose-timing interval, it can be supplemented and the next dose-timing should still be based on the original prescribed time; If the time of missing doses overruns more than half of the prescribed dose-timing interval, there is no need to supplement medications and the next dose-timing time should be advanced. |
| 3 | Do not share your ARV medications to others because each patient living with HIV has his or her own specific treatment plan and doses. |
| 4 | High level of ART adherence and optimal virological results are associated with the effectiveness of treatment. To ensure the effectiveness of ART, ≥95% of ART adherence should be guaranteed in a lifetime. |
| 5 | Your life depends on whether you can take ARV medications on schedule and at prescribed dose. |
| 6 | Some adverse effects may be reported during ART, most of which will wane over time. Some adverse effects can be alleviated by adjusting diet or other medications and gradually waned within 4-8 weeks after initiation of ART. |
| 7 | If you feel uncomfortable after taking ART medication, you should actively contact with the medical providers for help. You should not stop ART by yourself due to adverse reactions unless you have obtained permission from your medical providers. |
| 8 | If you do not follow the ART regime, the disease will continue to progress and your physical condition may deteriorate. This phenomenon may not occur immediately and take weeks, months or even years. You must keep daily taking ARV medication on time and at proper doses, and you cannot stop the medications without authorization. |
| 9 | Drug resistance refers to the decrease of sensitivity to ARV medications due to variation of virus gene. Taking medication on time can keep constant drug concentration in human body. Otherwise, the risk of drug resistance will increase. When the level of drug concentration in the blood is low, the virus will replicate furiously. |
| 10 | Do not stop taking ARV medications during travels/business travels. You can bring extra medications just in case. |
| 11 | Do not take medications with tea, milk or other drinks because some substances contained may interact with ARV medications, affecting its absorption and effectiveness, and even producing toxic effects. The correct way to take medications is to use boiled water with moderate temperature, or mineral water, purified water, etc. |
| 12 | Do not take tablets with dry swallowing, which will slow the dissolution of tablets and affect the efficacy. The correct way is as followed: taking a standing position, first putting the tablets into your mouth, then drinking a mouthful of water, slightly tilting your head up and swallowing. Do not lie down immediately after taking medications. Taking medications correctly can promote absorption, improve effects, and reduce the adverse reactions. |

**Table S2. HIV clinical messages**

| **Item** | **HIV clinical messages (delivered to intervention arm)** |
| --- | --- |
| 1 | HIV testing is the only scientific way to know whether you are infected with HIV including three steps: initially screening, re-testing and confirmatory testing. Rapid self-testing is initial screening of HIV antibody, which is influenced by many factors. It is recommended to go to local CDC for further consultation and confirmatory testing after self-testing regardless of negative or positive results. |
| 2 | The window period is referred to period from the entry of HIV into the human body to detection of HIV antibodies from blood. Although HIV antibody is not detected during window period, the virus has been in human body and can be detected by HIV RNA testing. Therefore, PLWH in the window period are also contagious. |
| 3 | Since 2016, ART standard in China has been adjusted to "Treat all". That is, no matter CD4 T-cell counts, patients can come to designated hospital to seek ART as long as HIV infection is diagnosed. For those with acute infection, consistent antiviral treatment is strongly recommended due to its high infectivity. |
| 4 | Severe opportunistic infections should be treated before initiation of ART. Some unstable chronic diseases such as severe heart, brain, kidney diseases and HIV related tumors may affect the safety and adherence of ART for PLWH. In this case, PLWH should be treated these diseases first and then initiate ART. |
| 5 | CD4 T-cell is an important immune cell and the target cell attacked by HIV. The CD4 T-cell counts play an important role in judging immune function impairment and treatment effect. From medical report, we can also see a kind of CD8 T-cells, also known as cytotoxic T-cells, whose function is to destroy infected cells including CD4 T-cells infected by HIV. In this way, the ratio of CD4 to CD8 on the report will be inverted, that is, the ratio of CD4 to CD8 is less than 1. In people who are not infected with HIV, the ratio of the two T-cells is usually greater than 1. The absolute count of CD4 T-cells sometimes fluctuates physiologically, while the ratio of CD4 to CD8 is relatively stable. |
| 6 | Viral load (VL) is the number of viruses per milliliter of blood. After standardized treatment, VL will be lower than the detection limit, indicating that the virus has been effectively suppressed and the damage of the virus to body has been greatly reduced. However, undetectable viruses in blood still distribute in semen, vaginal secretions, bone marrow and internal organs, and may be transmitted to others through unsafe sex or sharing needles. |

**Table S3. Peer education stories**

| **Item** | **Peer education stories (delivered to intervention arm)** |
| --- | --- |
| 1 | Xiao Bei (pseudonym): When I was an undergraduate student, on a whim, I went to the CDC for a test. I was overwhelmed when hearing that the screening result was positive. Fortunately, with the help of CDC staff and volunteers, I took the national free ARV medication within one week after the diagnosis. Now I regularly visit the doctor and receive physical examination. I consistently take medication at 10 p.m. every day and never miss doses. Just like washing before going to bed at night, I have developed a habit and have taken ARV medication for more than a year. At present, I think I am in good health. Except taking medication every day, I am not different from others and even pay more attention to my health. The doctor told me that I can live as long as general people if I consistently take medication on time.  **Conclusion: From now on, we should cherish ourselves, consistently take medicine and have good attitudes. I think our life will be better and better.** |
| 2 | Xiao Chen (pseudonym): I work in a company and have a good life. Unexpectedly, I was diagnosed as HIV infection about two years ago, I did not want to believe that and was extremely resistant to receive ART. I felt in good health and did not need to take medication. However, I did not expect to get herpes zoster and pneumonia six months later. After that, I followed the doctor's advice, received national free ART and took medication on time every day. My health gradually improved after standardized treatment.  **Conclusion: Your health will gradually improve if you consist on taking medication on time every day. Taking medication has become my favorite thing to do every day.** |
| 3 | Xiao Wang (pseudonym): At that time (October 2018), I just graduated and was not very satisfactory to my work. I began to have symptom of diarrhea in November, at least four times a day. I went to the hospital to get medications for gastroenteritis and was put on a drip for three days. I continued to have a fever in December. The doctor advised me to take blood for HIV testing. The screening results were positive. Then I tested for CD4 T-cell and liver and kidney function. The CD4 T-cell is an immune cell, representing the level of human body's immunity. At that time, I was in the stage of acute infection. My CD4 T-cell counts were only 218, which was relatively low. The doctor told me that CD4 should be above 500. After treatment, my CD4 has returned to the normal level. At present, I am in good health without any diarrhea or fever.  **Conclusion: CD4 T-cell is an important immune cell in human body. The testing results play an important role in evaluating the effect of treatment and immune function. The number of CD4 cells in general adults is 500-1600/mm^3^. If CD4 T-cell counts among PLWH descend progressively or irregularly, it indicates that the immune system has been seriously damaged. When CD4 T-cell counts are less than 200/mm^3^, it indicates that a variety of opportunistic infections or tumors may be reported.** |
| 4 | Xiao Wu (pseudonym): I have received ART for three and a half years. When I initially started to take medication, I often missed doses due to irregular schedule. In early 2019, I began to report stomachache and repeated fever. I went to the hospital for examination and learned that I was infected with intestinal tuberculosis. The results showed that CD4 was only about 150 and the viral load (VL) was 300,000. The VL refers to the number of virus in the blood (the lower the better). If the VL continues to be undetectable, it indicates that the possibility of transmission is very low. The doctor told me that it is due to my long-term irregular treatment. Since then, I took medication on time every day. Now my VL has been undetectable and my health is getting better.  **Conclusion: VL is the number of viruses per milliliter of blood. The result of VL is showed by the number of HIV RNA copies per milliliter of blood. Generally, after receiving ART for 3-6 months, the VL should lower than the detection limit. If the VL in the plasma remains at an undetectable level** **for more than 6 months, the risk of HIV transmission is very low.** |
| 5 | Da Yong (pseudonym): At that time, my parents knew my situation and never abandoned me. And I also got the help of my friends. Thereafter, I quit my stable job and devoted myself to the work of HIV prevention and care. Since I started the work, my mobile phone was in 24-hour standby mode. As long as I was awake, I would reply to the patients and answer their telephone inquiries. I am also an infected man and more likely to know patients’ real needs, which can better help them. Some of PLWH come to consult for seeking treatment and some just sought companionship. Every time the patients send a message to tell me that their health have improved, I think my work is meaningful.  **Conclusion: “I am also an infected person. I am also in good health” I often use this phrase to encourage those who are in trouble. I hope I can help more people, and I hope that the public will understand AIDS and PLWH.** |
| 6 | Shuai Shuai (pseudonym): Taking ARV medications (TFV/Lamivudine/EFV) on time is something I have to do every day. I take it well according to the doctor's prescription. Now my CD4 has risen to 750, and VL was lower than the detection level. However, in the spring of two years ago, I lost my two-months ARV medications by accident. For fear of influencing treatment, I asked my friends for help and borrowed medications (zidovudine). I thought the effect of ARV medications were consistent, but after taking it for two weeks, I had serious symptoms such as vomiting, headache and fatigue. This seriously affected my life and I lost my job. Later, with the help of the doctor, I adjusted the treatment plan so that my health gradually improved. I must cherish my medication and do not casually change my prescription. Do not borrow your medications without knowing others’ prescriptions.  **Conclusion: Different ARV medications have different effects. The treatment effect of taking wrong medications is poor and there may be adverse reactions. Follow the prescriptions and take good care of your medications.** |

**Table S4. Health behavior and nutrition messages**

| **Item** | **Health behavior and nutrition messages (delivered to control arm)** |
| --- | --- |
| 1 | **What are the main route of HIV transmission among MSM?**  The HIV infection among MSM is mainly through sexual transmission such as unprotected anal sex and oral sex. In addition, multiple sexual partners, commercial sex, drug use and SM will increase the risk of HIV infection. |
| 2 | **Are people reporting anal sex more likely to be infected with HIV?**  Unprotected anal sex contributes the highest risk of HIV infection. The mucosal damage (including the damage invisible to the naked eye) can lead to HIV infection. In particular, the anorectal mucosa is a single-layer columnar epithelium (thin and delicate), under which there are abundant capillaries and target cells of HIV. The pathogens can invade from the damaged mucosal of anus or rectum and directly enter the blood. |
| 3 | **Can people reporting oral sex be infected with HIV?**  There is a lower risk of HIV infection of oral sex than anal sex. When oral mucous membrane is damaged, there may be risk of HIV infection for oral sex. In real life, it may be difficult to detect the small wound of oral mucosa. In order to minimize the risk of HIV infection, it is recommended to use condoms during oral sex. |
| 4 | **Can semen shoot on the face cause HIV infection?**  The virus cannot cause infection through facial skin with no breakage, but exposure of facial skin with breakage (such as pimples, acne, scratches, etc.) or eye mucosa to semen can increase the risk of HIV infection. |
| 5 | **Regular screening and receiving diagnosis and treatment of sexually transmitted diseases (STD) can reduce the risk of HIV infection.**  The risk of HIV infection is much higher among STD patients. This is because infection of STD can cause genital inflammation or ulcers, which makes it easier for HIV to invade. The risk of HIV infection is especially higher among those infected with STD such as syphilis, genital herpes and chancre. Regular screening and receiving standardized treatment in authoritative institutions will reduce the risk of HIV infection. |
| 6 | **HIV testing is the only way to judge whether a person is infected with HIV instead of appearances.**  The incubation period of PLWH can be up to 8-10 years. During the incubation period, we cannot judge whether a person is infected with HIV through appearance. Simultaneously, some symptoms are not unique to AIDS. Therefore, HIV infection cannot be judged by appearance or symptoms. If you want to know whether you are infected with HIV, testing is the only way. |
| 7 | **You should disclose your HIV status to your sexual partners once diagnosed with HIV. It is neither moral or legal to deliberately concealing and transmitting HIV**  PLWH are obliged to disclose their infection status to sexual partners and regulate their behaviors to avoid HIV transmission. Deliberately concealing and transmitting HIV is immoral. Simultaneously, it also violates Chinese national laws and regulations. Those who deliberately transmitting HIV need to bear corresponding legal responsibilities. |
| 8 | **Consistent use of condoms during sexual behavior can effectively reduce the risk of infection and transmission of HIV and STDs**  Consistent use of condoms can play a physical barrier role in homosexual anal sex by preventing virus in semen or prostate fluid from entering the body through anorectal mucosa. The effectiveness of condoms in the prevention of STDs and HIV transmission has been proved by a large number of scientific research and long-term work of disease prevention and control. |
| 9 | **Being away from illicit drugs, especially new-type illicit drugs (such as RUSH, zero capsule, etc.) can reduce the risk of HIV infection.**  Abusing new-type illicit drugs can inhibit or excite people's central nervous system, causing hallucination and increasing the possibility of high-risk sexual behavior such as condomless anal sex. Similarly, some drugs (such as rush, etc.) can increase heart rate and blood pressure, cause redness and fever, produce physiological feelings similar to orgasm, and increase the risk of high-risk sexual behavior. In addition, frequent use of Rush can damage your health. Take the initiative to stay away from new-type illicit drugs. |
| 10 | **How to choose lubricating oil?**  The lubricants need to be used in anal sex because anus and intestines cannot secrete enough body fluid to lubricate. However, oily substances can make the condom be brittle, leading to the condom easy to break during anal sex. Therefore, it is not recommended to use oily substances (such as Vaseline, baby oil, bath liquid and massage oil) as anal sex lubricants. Water soluble lubricant is more suitable for MSM during anal sex. |
| 11 | **How to adjust diet when nausea and vomiting?**  A small amount of low-fat food can inhibit nausea and vomiting; Try to eat dry food (such as dry bread and biscuits); Eat salty food, not sweets; Drink clear soup instead of greasy soup; Don't lie down immediately after dinner, preferably an hour apart; A small number of meals should be taken, and patients can eat more when they feel better; Avoid spicy, high-fat and caffeinated foods. |
| 12 | **How to adjust diet when diarrhea?**  Eat low-fat foods and prohibit greasy foods (such as fried foods); High protein and high energy diet (such as seafood and bean products such as fish and shrimp); Avoid eating more fiber (such as leek and celery) and rough food (such as sausage and bacon); Supplement vitamins and minerals. When diarrhea is serious, it is preferable to eat less vegetables and fruits and supplement vitamins through fresh fruit juice, tomato juice, etc; Replenish sufficient water (drink 3-4 cups more water than usual) and substances rich in potassium ions (such as bananas and potatoes); Spicy, smoking and drinking are forbidden. Do not use milk. |
