# Supplementary material for: A differentiated digital intervention to improve antiretroviral therapy adherence among men who have sex with men living with HIV in China: a randomized controlled trial
Source: BMC Med. 2022 Oct 10;20:341. doi: 10.1186/s12916-022-02538-3 (PMC9549628; doi:10.1186/s12916-022-02538-3)
Supplement: Supplementary file 4 — Additional file 4. Short articles shared to QQ group. [file 12916_2022_2538_MOESM4_ESM.docx]

**Additional file 4**

**Short articles shared to QQ group**

| **Intervention Time** | **Themes of short articles shared to QQ group** |
| --- | --- |
| Week 1 | The significant progress in the field of HIV/AIDS in 2020 |
| Week 2 | Individualized choice of antiretroviral medication based on the updated ART guidelines released by EACS in 2020 |
| Week 3 | Important tips for PLWH who are on ART during COVID-19 pandemic (released by China CDC) |
| Week 4 | Can PLWH be vaccinated against COVID-19? (released by UNAIDS) |
| Week 5 | Whether there is no need to worry if U=U |
| Week 6 | Side effects and management of EFV (efavirenz) |
| Week 7 | How to take LPV/r properly? |
| Week 8 | Five suggestions for PLWH during the Spring Festival of China (released by local CDC) |
| Week 9 | Address and contact information of all ART institutions in China, attached query links (released by China CDC) |
| Week 10 | From a gay living with HIV for ten years: how do I deal with discrimination |
| Week 11 | Why do PLWH need to take ARV medications on time |
| Week 12 | Gilead and Merck announced a partnership to develop long-term HIV treatment programs |
| Week 13 | Is long-term low CD4 T-cell counts regarded ART failure? Is it necessary to change ART medications? (released by local CDC) |
| Week 14 | “Mosaic” HIV-1 vaccine——new hope for HIV/AIDS vaccine (released by China CDC) |
| Week 15 | Dietary guidelines for PLWH |
| Week 16 | Tuberculosis——the biggest killer for death of people living with HIV (released by UNAIDS) |
| Week 17 | Ten tips about drug resistance of ART |
| Week 18 | Expert suggestions about COVID-19 vaccine for PLWH (released by Chinese Medical Association) |
| Week 19 | Benefits of exercising for PLWH. Recommendations for the ways of exercise and notes |
| Week 20 | How many kinds of ARV medications in total |
| Week 21 | The mechanism influencing ART effectiveness and side effects |
| Week 22 | Is it necessary to take ARV medication punctually to the minute? (released by local CDC) |
| Week 23 | How far are we from HIV vaccine? (released by UNAIDS) |
| Week 24 | Global AIDS strategies in 2021-2026 (released by UNAIDS) |
