# Supplementary material for: A differentiated digital intervention to improve antiretroviral therapy adherence among men who have sex with men living with HIV in China: a randomized controlled trial
Source: BMC Med. 2022 Oct 10;20:341. doi: 10.1186/s12916-022-02538-3 (PMC9549628; doi:10.1186/s12916-022-02538-3)
Supplement: Supplementary file 5 — Additional file 5. Basic characteristics of the participants in three digital subgroups. Table S1. Socio-demographic, behavioral and clinical characteristics of the study participants in three digital subgroups. [file 12916_2022_2538_MOESM5_ESM.docx]

**Additional file 5**

**Basic characteristics of the participants in three digital subgroups**

Table S1 Socio-demographic, behavioral and clinical characteristics of the study participants in three digital subgroups

| **Characteristics** | **Text message subgroup** | | | **Instant message subgroup** | | | **Instant message plus social media subgroup** | | |
| --- | --- | --- | --- | --- | --- | --- | --- | --- | --- |
|  | **Total (n=144)** | **Intervention group (n=72)** | **Control group (n=72)** | **Total (n=290)** | **Intervention group (n=145)** | **Control group (n=145)** | **Total (n=142)** | **Intervention group (n=71)** | **Control group (n=71)** |
| **Age, years** |  |  |  |  |  |  |  |  |  |
| ≤30 | 44 (30.6) | 23 (31.9) | 21 (29.2) | 113 (39.0) | 52 (35.9) | 61 (42.1) | 57 (40.1) | 26 (36.6) | 31 (43.7) |
| 31-40 | 65 (45.1) | 30 (41.7) | 35 (48.6) | 119 (41.0) | 65 (44.8) | 54 (37.2) | 62 (43.7) | 33 (46.5) | 29 (40.8) |
| >40 | 35 (24.3) | 19 (26.4) | 16 (22.2) | 58 (20.0) | 28 (19.3) | 30 (20.7) | 23 (16.2) | 12 (16.9) | 11 (15.5) |
| **Education level** |  |  |  |  |  |  |  |  |  |
| High school or below | 66 (45.8) | 34 (47.2) | 32 (44.4) | 97 (33.4) | 50 (34.5) | 47 (32.4) | 59 (41.8) | 34 (48.6) | 25 (35.2) |
| College or beyond | 78 (54.2) | 38 (52.8) | 40 (55.6) | 193 (66.6) | 95 (65.5) | 98 (67.6) | 82 (58.2) | 36 (51.4) | 46 (64.8) |
| **Annual income, USD** |  |  |  |  |  |  |  |  |  |
| ≤9474 | 77 (53.5) | 40 (55.6) | 37 (51.4) | 133 (45.9) | 72 (49.7) | 61 (42.7) | 66 (46.5) | 36 (50.7) | 30 (42.3) |
| 9475-15158 | 38 (26.4) | 18 (25.0) | 20 (27.8) | 87 (30.0) | 42 (29.0) | 45 (31.0) | 52 (36.6) | 22 (31.0) | 30 (42.3) |
| >15158 | 29 (20.1) | 14 (19.4) | 15 (20.8) | 70 (24.1) | 31 (21.4) | 39 (26.9) | 24 (16.9) | 13 (18.3) | 11 (15.5) |
| **Marital status** |  |  |  |  |  |  |  |  |  |
| Unmarried/divorced/  widowed | 108 (75.0) | 52 (72.2) | 56 (77.8) | 235 (81.0) | 108 (74.5) | 127 (87.6) | 122 (85.9) | 61 (85.9) | 61 (85.9) |
| Married/cohabitating | 36 (25.0) | 20 (27.8) | 16 (22.2) | 55 (19.0) | 37 (25.5) | 18 (12.4） | 20 (14.1) | 10 (14.1) | 10 (14.1) |
| **Sexual orientation** |  |  |  |  |  |  |  |  |  |
| Gay | 87 (60.4) | 42 (58.3) | 45 (62.5) | 182 (62.8) | 86 (59.3) | 96 (66.2) | 79 (55.6) | 36 (50.7) | 43 (60.6) |
| Bisexual | 37 (25.7) | 20 (27.8) | 17 (23.6) | 76 (26.2) | 39 (26.9) | 37 (25.5) | 38 (26.8) | 21 (29.6) | 17 (23.9) |
| Heterosexual or others | 20 (13.9) | 10 (13.9) | 10 (13.9) | 32 (11.0) | 20 (13.8) | 12 (8.3) | 25 (17.6) | 14 (19.7) | 11 (15.5) |
| **Sexual orientation disclosure^†^** |  |  |  |  |  |  |  |  |  |
| Yes | 82 (56.9) | 43 (59.7) | 39 (54.2) | 158 (54.5) | 82 (56.6) | 76 (52.4) | 82 (57.7) | 44 (62.0) | 38 (53.5) |
| No | 62 (43.1) | 29 (40.3) | 33 (45.8) | 132 (45.5) | 63 (43.4) | 69 (47.6) | 60 (42.3) | 27 (38.0) | 33 (46.5) |
| **HIV status disclosure^‡^** |  |  |  |  |  |  |  |  |  |
| Yes | 92 (63.9) | 48 (66.7) | 44 (61.1) | 178 (61.4) | 92 (63.4) | 86 (59.3) | 89 (62.7) | 41 (57.7) | 48 (67.6) |
| No | 52 (36.1) | 24 (33.3) | 28 (38.9) | 112 (38.6) | 53 (36.6) | 59 (40.7) | 53 (37.3) | 30 (42.3) | 23 (32.4) |
| **Alcohol use in the past 12 months** |  |  |  |  |  |  |  |  |  |
| Yes | 88 (61.1) | 41 (56.9) | 47 (65.3) | 163 (56.2) | 80 (55.2) | 83 (57.2) | 85 (59.9) | 42 (59.2) | 43 (60.6) |
| No | 56 (38.9) | 31 (43.1) | 25 (34.7) | 127 (43.8) | 65 (44.8) | 62 (42.8) | 57 (40.1) | 29 (40.8) | 28 (39.4) |
| **Viral suppression^§^** |  |  |  |  |  |  |  |  |  |
| Yes | 103 (82.4) | 50 (78.1) | 53 (86.9) | 214 (82.3) | 104 (80.0) | 110 (84.6) | 115 (91.3) | 55 (90.2) | 60 (92.3) |
| No | 22 (17.6) | 14 (21.9) | 8 (13.1) | 46 (17.7) | 26 (20.0) | 20 (15.4) | 11 (8.7) | 6 (9.8) | 5 (7.7) |
| **CD4 T-cell counts, M±IQR** | 581.00±367.00 | 586.00±394.00 | 574.00±402.50 | 582.00±401.00 | 577.00±440.00 | 594.50±355.50 | 598.00±370.00 | 656.00±391.50 | 378.00±329.00 |
| **Duration of ART, years** |  |  |  |  |  |  |  |  |  |
| ≤3 | 85 (59.0) | 42 (58.3) | 43 (59.7) | 178 (61.4) | 94 (64.8) | 84 (57.9) | 85 (59.9) | 41 (57.7) | 44 (62.0) |
| >3 | 59 (41.0) | 30 (41.7) | 29 (40.3) | 112 (38.6) | 51 (35.2) | 61 (42.1) | 57 (40.1) | 30 (42.3) | 27 (38.0) |
| **Frequency of medication-taking** |  |  |  |  |  |  |  |  |  |
| Once a day | 84 (58.3) | 38 (52.8) | 46 (63.9) | 170 (58.8) | 87 (60.4) | 83 (57.2) | 77 (54.2) | 40 (56.3) | 37 (52.1) |
| Twice a day | 60 (41.7) | 34 (47.2) | 26 (36.1) | 119 (41.2) | 57 (39.6) | 62 (42.8) | 65 (45.8) | 31 (43.7) | 34 (47.9) |
| **Regime of ART** |  |  |  |  |  |  |  |  |  |
| Three | 132 (91.7) | 67 (93.1) | 65 (90.3) | 268 (92.4) | 132 (91.0) | 136 (93.8) | 131 (92.3) | 66 (93.0) | 65 (91.5) |
| Two or one | 12 (8.3) | 5 (6.9) | 7 (9.7) | 22 (7.6) | 13 (9.0) | 9 (6.2) | 11 (7.7) | 5 (7.0) | 6 (8.5) |
| **Received adherence education before ART initiation** |  |  |  |  |  |  |  |  |  |
| Yes | 139 (96.5) | 69 (95.8) | 70 (97.2) | 271 (93.4) | 136 (93.8) | 135 (93.1) | 135 (95.1) | 69 (97.2) | 66 (93.0) |
| No | 5 (3.5) | 3 (4.2) | 2 (2.8) | 19 (6.6) | 9 (6.2) | 10 (6.9) | 7 (4.9) | 2 (2.8) | 5 (7.0) |
| **Used medication reminders** |  |  |  |  |  |  |  |  |  |
| Yes | 135 (93.8) | 67 (93.1) | 68 (94.4) | 260 (89.7) | 130 (89.7) | 130 (89.7) | 132 (93.0) | 64 (90.1) | 68 (95.8) |
| No | 9 (6.2) | 5 (6.9) | 4 (5.6) | 30 (10.3) | 15 (10.3) | 15 (10.3) | 10 (7.0) | 7 (9.9) | 3 (4.2) |
| **Ever experienced any side effects during ART** |  |  |  |  |  |  |  |  |  |
| No | 58 (40.3) | 30 (41.7) | 28 (38.9) | 107 (36.9) | 53 (36.6) | 54 (37.2) | 54 (38.0) | 25 (35.2) | 29 (40.8) |
| Yes | 86 (59.7) | 42 (58.3) | 44 (61.1) | 183 (63.1) | 92 (63.4) | 91 (62.8) | 88 (62.0) | 46 (64.8) | 42 (59.2) |
| Data are presented as no. (%) unless otherwise indicated. Missing values were not taken into account in the percentage calculation.  Abbreviations: ART, antiretroviral therapy; USD, United States Dollars, IQR, interquartile range  ^†^ Has told anyone (except homosexual partner) about sexual orientation or sexual history with men  ^‡^ Has told anyone (except health providers in designated ART sites) about HIV positive-status  ^§^ Defined as undetectable viral load (i.e., <20 copies/ml) | | | | | | | | | |
