# Supplementary material for: A differentiated digital intervention to improve antiretroviral therapy adherence among men who have sex with men living with HIV in China: a randomized controlled trial
Source: BMC Med. 2022 Oct 10;20:341. doi: 10.1186/s12916-022-02538-3 (PMC9549628; doi:10.1186/s12916-022-02538-3)
Supplement: Supplementary file 6 — Additional file 6. Measurements of the secondary outcomes at baseline and two follow-ups. Table S1. Measurements of CD4 T cell counts and proportions of HIV viral suppression at baseline and two follow-ups. Table S2. Measurements of HIV treatment adherence self-efficacy at baseline and two follow-ups. Table S3. Measurements of quality of life at baseline and two follow-ups. [file 12916_2022_2538_MOESM6_ESM.docx]

**Additional file 6**

**Measurements of secondary outcomes at baseline and two follow-ups**

Table S1 Measurements of CD4 T-cell counts and proportions of HIV viral suppression at baseline and two follow-ups

|  | **CD4 T-cell counts**^†^ | | **Proportions of viral suppression**^‡^ | |
| --- | --- | --- | --- | --- |
| **Group** | **Pre-intervention** | **Post-intervention** | **Pre-intervention** | **Post-intervention** |
| **Total** |  |  |  |  |
| Intervention arm | 586.00± 434.00 | 586.00± 387.00. | 82.0% (209/255) | 94.5% (260/275) |
| Control arm | 583.00± 352.50 | 582.00± 331.00 | 87.1% (223/256) | 93.0% (253/272) |
| **Text message subgroup** |  |  |  |  |
| Intervention group | 586.00± 394.00 | 535.50± 350.00 | 78.1% (50/64) | 97.1% (67/69) |
| Control group | 574.00± 402.50 | 593.00± 345.50 | 86.9% (53/61) | 97.1% (66/68) |
| **Instant message subgroup** |  |  |  |  |
| Intervention group | 577.00± 440.00 | 594.00± 392.00 | 80.0% (104/130) | 93.6% (131/140) |
| Control group | 594.50± 355.50 | 593.00± 345.00 | 84.6% (110/130) | 93.4% (127/136) |
| **Instant message plus social media subgroup** |  |  |  |  |
| Intervention group | 656.00± 391.50 | 633.00± 322.00 | 90.2% (55/61) | 93.9% (62/66) |
| Control group | 578.00± 329.00 | 558.00± 307.00 | 92.3% (60/65) | 88.2% (60/68) |
| ^†^ Data are presented as M±IQR (IQR, interquartile range)  ^‡^ Data are presented as % | | | | |

Table S2 Measurements of HIV treatment adherence self-efficacy at baseline and two follow-ups

| **Group** | **Baseline** | **First follow-up** | **Second follow-up** | |
| --- | --- | --- | --- | --- |
| **Total** |  |  |  |  |
| Intervention arm | 114.48±15.38 | 114.68±10.90 | 116.73±7.60 |  |
| Control arm | 114.49±14.86 | 111.84±19.32 | 113.62±13.56 |  |
| **Text message subgroup** |  |  |  |  |
| Intervention group | 114.53±10.36 | 114.60±10.68 | 116.13±7.00 |  |
| Control group | 112.72±16.77 | 110.45±19.11 | 113.11±11.97 |  |
| **Instant message subgroup** |  |  |  |  |
| Intervention group | 115.38±13.71 | 114.50±10.83 | 117.09±7.88 |  |
| Control group | 115.50±9.74 | 113.25±15.60 | 112.91±14.65 |  |
| **Instant message plus social media subgroup** |  |  |  |  |
| Intervention group | 112.61±21.64 | 115.13±11.39 | 116.62±7.70 |  |
| Control group | 112.99±20.43 | 110.46±25.21 | 115.47±12.82 |  |
| Data are presented as Mean±SD (SD, standard deviation) | | | |  |

Table S3 Measurements of quality of life at baseline and two follow-ups

| **Group** | **Baseline** | **First follow-up** | **Second follow-up** |
| --- | --- | --- | --- |
| **Total** |  |  |  |
| Intervention arm | 88.20±12.50 | 88.84±13.23 | 89.97±13.58 |
| Control arm | 87.42±13.05 | 88.23±14.04 | 87.05±14.14 |
| **Text message subgroup** |  |  |  |
| Intervention group | 89.36±11.96 | 90.92±13.17 | 90.34±12.77 |
| Control group | 86.27±13.68 | 90.58±12.94 | 89.16±13.44 |
| **Instant message subgroup** |  |  |  |
| Intervention group | 88.13±12.46 | 87.73±12.59 | 89.76±13.43 |
| Control group | 88.53±13.04 | 88.23±14.49 | 86.47±14.38 |
| **Instant message plus social media subgroup** |  |  |  |
| Intervention group | 87.17±13.17 | 89.13±14.51 | 89.99±15.00 |
| Control group | 86.30±12.37 | 85.98±14.00 | 86.16±14.33 |
| Data are presented as Mean±SD (SD, standard deviation) | | | |
