# Supplementary material for: A differentiated digital intervention to improve antiretroviral therapy adherence among men who have sex with men living with HIV in China: a randomized controlled trial
Source: BMC Med. 2022 Oct 10;20:341. doi: 10.1186/s12916-022-02538-3 (PMC9549628; doi:10.1186/s12916-022-02538-3)
Supplement: Supplementary file 7 — Additional file 7. Sensitivity analysis of combining two subgroups. Figure S1. Proportions of optimal ART adherence in the combined subgroups (i.e., instant message subgroup and instant message plus social media subgroup). Figure S2. Effect of the combined instant message-based and instant message plus social media intervention on ART adherence among MSM living with HIV in China, 2020-2021. Abbreviations: ART, antiretroviral therapy; RR, risk ratio; CI, confidence interval; ITT, intention-to-treat; PP, per-protocol; AT, as-treated. [file 12916_2022_2538_MOESM7_ESM.docx]

**Additional file 7**

**Sensitivity analysis of combining two subgroups**

Given baseline differences in the ART adherence in the instant message subgroup, we conducted sensitivity analysis by combining two subgroups (i.e., instant message subgroup and instant message plus social media subgroup) to evaluate the effectiveness of instant message-based intervention.

As displayed in Figure S1, at baseline, the proportion of optimal ART adherence was 78.7% in the intervention group and 72.7% in the control group. At the second follow-up (post-intervention), the proportion of optimal ART adherence was 85.1% in the intervention group and 71.0% in the control group.

As displayed in Figure S2, after combining the instant message subgroup and instant message plus social media subgroup, the intervention was effective in improving ART adherence (ITT: RR=1.81, 95%CI: 1.16-2.82; PP analysis: RR=2.52, 95%CI 1.52-4.17; AT analysis: RR=2.46, 95%CI 1.55-3.88).


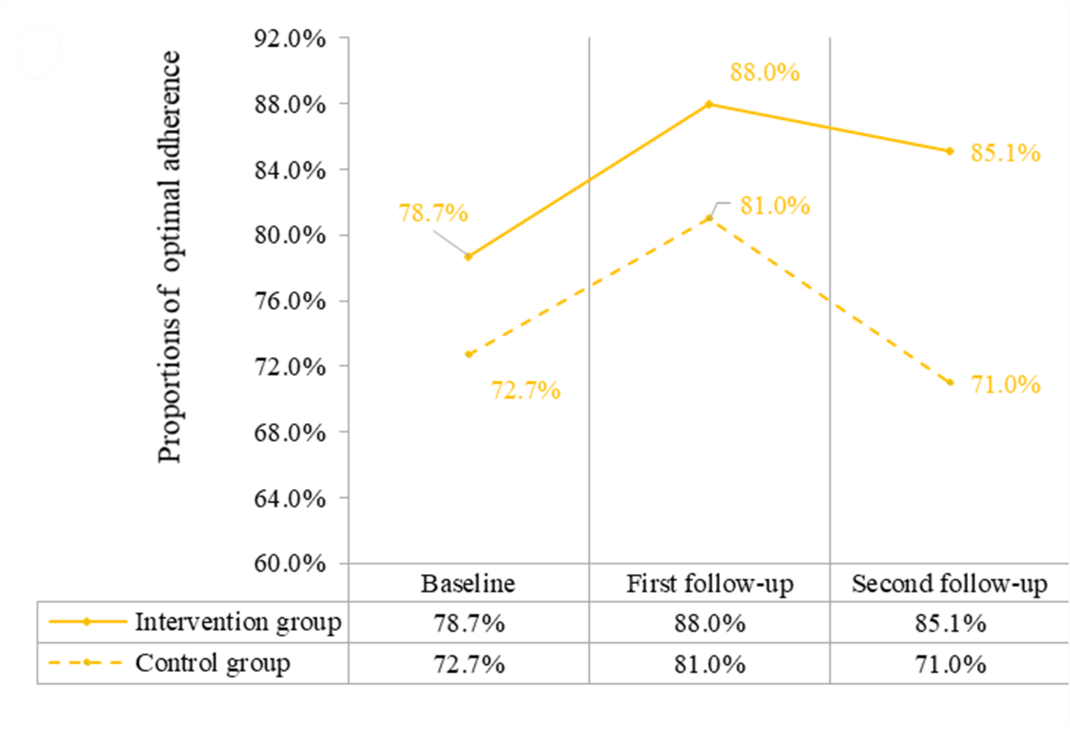


Figure S1 Proportions of optimal ART adherence in the combined subgroups (i.e., instant message subgroup and instant message plus social media subgroup)


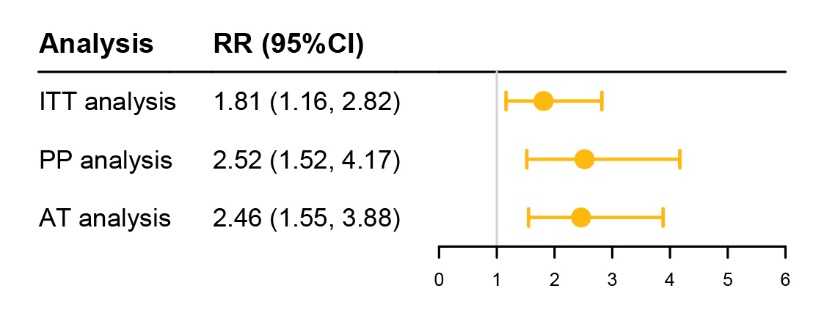


Figure S2 Effect of the combined instant message-based and instant message plus social media intervention on ART adherence among MSM living with HIV in China, 2020-2021

Abbreviations: ART, antiretroviral therapy; RR, risk ratio; CI, confidence interval; ITT, intention-to-treat; PP, per-protocol; AT, as-treated
