# Supplementary material for: A differentiated digital intervention to improve antiretroviral therapy adherence among men who have sex with men living with HIV in China: a randomized controlled trial
Source: BMC Med. 2022 Oct 10;20:341. doi: 10.1186/s12916-022-02538-3 (PMC9549628; doi:10.1186/s12916-022-02538-3)
Supplement: Supplementary file 8 — Additional file 8. Absolute effect size of primary outcome. Table S1. Absolut effect size of differentiated digital intervention on ART adherence among MSM living with HIV in China, 2020-2021. [file 12916_2022_2538_MOESM8_ESM.docx]

**Additional file 8**

**Absolute effect size of primary outcome**

Table S1 Absolut effect size of differentiated digital intervention on ART adherence among MSM living with HIV in China, 2020-2021

| **Analysis** | **Effect** | **RD, percent (95%CI)** |
| --- | --- | --- |
| ITT analysis | Differentiated digital intervention | 11.8 (4.5, 19.1) |
|  | By ART duration |  |
|  | <=3 years | 13.4 (4.4, 22.4) |
|  | >3 years | 8.9 (-3.3, 21.1) |
|  | By digital strategy |  |
|  | Text message-based intervention | 5.2 (-10.0, 20.4) |
|  | Instant message-based intervention | 16.9 (6.9, 26.9) |
|  | Instant message plus social media intervention | 7.6 (-7.1, 22.3) |
| PP analysis | Differentiated digital intervention | 14.8 (6.8, 22.8) |
|  | By ART duration |  |
|  | <=3 years | 15.8 (5.8, 25.8) |
|  | >3 years | 13.1 (-0.1, 26.3) |
|  | By digital strategy |  |
|  | Text message-based intervention | -1.8 (-19.8, 16.2) |
|  | Instant message-based intervention | 22.7 (11.7, 33.7) |
|  | Instant message plus social media intervention | 13.3 (-1.8, 28.4) |
| AT analysis | Differentiated digital intervention | 13.7 (6.4, 21.0) |
|  | By ART duration |  |
|  | <=3 years | 13.6 (4.5, 22.7) |
|  | >3 years | 13.5 (1.4, 25.6) |
|  | By digital strategy |  |
|  | Text message-based intervention | -6.1 (-22.2, 10.0) |
|  | Instant message-based intervention | 21.9 (11.5, 32.3) |
|  | Instant message plus social media intervention | 15.7 (1.3, 30.1) |
| Abbreviation: ITT, intention-to-treat; PP, per-protocol; AT, as-treated; ART, antiretroviral therapy; MSM, men who have sex with men; RD, risk difference; CI, confidence interval | | |
