# Supplementary material for: A differentiated digital intervention to improve antiretroviral therapy adherence among men who have sex with men living with HIV in China: a randomized controlled trial
Source: BMC Med. 2022 Oct 10;20:341. doi: 10.1186/s12916-022-02538-3 (PMC9549628; doi:10.1186/s12916-022-02538-3)
Supplement: Supplementary file 9 — Additional file 9. Intervention effect on various domains of quality of life using ITT analysis. Table S1. Effect of differentiated digital intervention on various domains of quality of life using ITT analysis among MSM living with HIV in China, 2020-2021. [file 12916_2022_2538_MOESM9_ESM.docx]

**Additional file 9**

**Intervention effect on various domains of quality of life using ITT analysis**

Table S1 Effect of differentiated digital intervention on various domains of quality of life using ITT analysis among MSM living with HIV in China, 2020-2021

|  | **Differentiated digital intervention (Total)** | **Text message-based intervention** | **Instant message-based intervention** | **Instant message plus social media intervention** |
| --- | --- | --- | --- | --- |
| **Outcomes** | **MD (95%CI)** | | | |
| Physical domain of QoL | 0.08 (-0.13, 0.28) | -0.03 (-0.40, 0.35) | 0.04 (-0.26, 0.33) | 0.27 (-0.15, 0.69) |
| Psychological domain of QOL | 0.12 (-0.10, 0.35) | -0.17 (-0.65, 0.31) | 0.03 (-0.28, 0.34) | 0.64 (0.16, 1.11) |
| Independence domain of QOL | 0.13 (-0.05, 0.31) | -0.05 (-0.37, 0.28) | 0.26 (<-0.01, 0.53) | 0.01 (-0.37, 0.39) |
| Social relationship domain of QOL | 0.10 (-0.13, 0.33) | -0.34 (-0.75, 0.08) | 0.25 (-0.08, 0.58) | 0.24 (-0.26, 0.74) |
| Environmental domain of QOL | 0.12 (-0.10, 0.33) | -0.22 (-0.69, 0.26) | 0.22 (-0.08, 0.52) | 0.24 (-0.20, 0.69) |
| Spiritual domain of QOL | -0.08 (-0.37, 0.21) | -0.70 (-1.34, -0.06) | 0.06 (-0.34, 0.46) | 0.26 (-0.30, 0.82) |
| Abbreviations: ITT, intention-to-treat; QoL, quality of life; MD, mean difference; CI, confidence interval | | | | |
