# Supplementary material for: A differentiated digital intervention to improve antiretroviral therapy adherence among men who have sex with men living with HIV in China: a randomized controlled trial
Source: BMC Med. 2022 Oct 10;20:341. doi: 10.1186/s12916-022-02538-3 (PMC9549628; doi:10.1186/s12916-022-02538-3)
Supplement: Supplementary file 10 — Additional file 10. Sensitivity analyses of the secondary outcomes. Table S1. Effect of differentiated digital intervention on secondary outcomes using PP analysis among MSM living with HIV in China, 2020-2021. Table S2. Effect of differentiated digital intervention on secondary outcomes using AT analysis among MSM living with HIV in China, 2020-2021. [file 12916_2022_2538_MOESM10_ESM.docx]

**Additional file 10**

**Sensitivity analyses of the secondary outcomes**

Table S1. Effect of differentiated digital intervention on secondary outcomes using PP analysis among MSM living with HIV in China, 2020-2021

|  | **Differentiated digital intervention (Total)** | **Text message-based intervention** | **Instant message-based intervention** | **Instant message plus social media intervention** |
| --- | --- | --- | --- | --- |
| **Outcomes** | **RR (95%CI)** | | | |
| Viral suppression^§^ | 1.27 (0.51, 3.12) | 0.94 (0.11, 8.18) | 1.55^†^ (<0.01, >99.99) | 3.32 (0.61, 17.99) |
|  | **MD (95%CI)** | | | |
| CD4 T-cell counts | 1.17 (-15.68, 18.02) | -5.91 (-43.32, 31.51) | 14.78 (-8.32, 37.87) | -20.44 (-52.94, 12.06) |
| HIV treatment self-efficacy | 1.84 (0.02, 3.67) | 0.61 (-2.67, 3.90) | 2.22 (-0.06, 4.50) | 2.21 (-2.54, 6.95) |
| QoL | 0.51 (-0.53, 1.54) | -0.84 (-2.85, 1.17) | 0.75 (-0.75, 2.24) | 1.30 (-0.77, 3.37) |
| Physical domain of QoL | 0.14 (-0.10, 0.37) | 0.13 (-0.31, 0.56) | 0.10 (-0.24, 0.44) | 0.22 (-0.27, 0.71) |
| Psychological domain of QOL | 0.09 (-0.17, 0.36) | -0.28 (-0.83, 0.28) | 0.03 (-0.33, 0.40) | 0.56 (0.01, 1.12) |
| Independence domain of QOL | 0.17 (-0.03, 0.37) | 0.15 (-0.22, 0.51) | 0.27 (-0.02, 0.57) | -0.02 (-0.47, 0.42) |
| Social relationship domain of QOL | 0.14 (-0.12, 0.41) | -0.10 (-0.59, 0.38) | 0.23 (-0.16, 0.61) | 0.22 (-0.35, 0.78) |
| Environmental domain of QOL | 0.11 (-0.15, 0.36) | -0.22 (-0.76, 0.32) | 0.21 (-0.15, 0.56) | 0.21 (-0.28, 0.71) |
| Spiritual domain of QOL | -0.14 (-0.49, 0.21) | -0.51 (-1.31, 0.29) | -0.09 (-0.57, 0.38) | 0.11 (-0.56, 0.78) |
| Abbreviation: PP, per-protocol; ART, antiretroviral therapy; QoL, quality of life; MSM, men who have sex with men; RR, risk ratio; MD, mean difference; CI, confidence interval  ^†^ Using log Poisson GLMM due to non-convergence phenomenon  ^§^ Defined as undetectable viral load (i.e., <20 copies/ml) | | | | |

Table S2. Effect of differentiated digital intervention on secondary outcomes using AT analysis among MSM living with HIV in China, 2020-2021

|  | **Differentiated digital intervention (Total)** | **Text message-based intervention** | **Instant message-based intervention** | **Instant message plus social media intervention** |
| --- | --- | --- | --- | --- |
| **Outcomes** | **RR (95%CI)** | | | |
| Viral suppression^§^ | 1.27 (0.56, 2.89) | 0.72 (0.09, 6.06) | 1.06 (0.30, 3.76) | 2.53 (0.57, 11.29) |
|  | **MD (95%CI)** | | | |
| CD4 T-cell counts | -2.85 (-19.54, 13.83) | -3.47 (-35.51, 28.56) | 13.54 (-7.26, 34.33) | -32.23 (-73.37, 8.91) |
| HIV treatment self-efficacy | 1.57 (-0.11, 3.24) | -0.06 (-3.21, 3.08) | 1.92 (-0.21, 4.05) | 2.40 (-1.79, 6.59) |
| QoL | 0.41 (-0.56, 1.38) | 0.09 (-1.81, 2.00) | 0.21 (-1.20, 1.61) | 1.14 (-0.78, 3.06) |
| Physical domain of QoL | 0.19 (-0.03, 0.41) | 0.27 (-0.14, 0.69) | 0.08 (-0.24, 0.40) | 0.32 (-0.14, 0.77) |
| Psychological domain of QOL | 0.08 (-0.17, 0.32) | -0.23 (-0.75, 0.29) | 0.03 (-0.30, 0.37) | 0.47 (-0.04, 0.98) |
| Independence domain of QOL | 0.11 (-0.09, 0.30) | 0.25 (-0.11, 0.61) | 0.12 (-0.16, 0.40) | -0.05 (-0.47, 0.36) |
| Social relationship domain of QOL | 0.14 (-0.11, 0.39) | 0.11 (-0.35, 0.56) | 0.12 (-0.24, 0.47) | 0.21 (-0.31, 0.74) |
| Environmental domain of QOL | 0.06 (-0.17, 0.30) | -0.12 (-0.63, 0.39) | 0.08 (-0.25, 0.41) | 0.21 (-0.26, 0.68) |
| Spiritual domain of QOL | -0.16 (-0.49, 0.16) | -0.18 (-0.91, 0.54) | -0.22 (-0.66, 0.22) | -0.02 (-0.63, 0.59) |
| Abbreviation: AT, as-treated; ART, antiretroviral therapy; QoL, quality of life; MSM, men who have sex with men; RR, risk ratio; MD, mean difference; CI, confidence interval  ^§^ Defined as undetectable viral load (i.e., <20 copies/ml) | | | | |
